# Whole chloroplast genome and gene locus phylogenies reveal the taxonomic placement and relationship of *Tripidium* (Panicoideae: Andropogoneae) to sugarcane

**DOI:** 10.1186/s12862-019-1356-9

**Published:** 2019-01-25

**Authors:** Dyfed Lloyd Evans, Shailesh V. Joshi, Jianping Wang

**Affiliations:** 1 South African Sugarcane Research Institute, 170 Flanders Drive, Private Bag X02, Mount Edgecombe, Durban, 4300 South Africa; 2 School of Life Sciences, College of Agriculture, Engineering and Science, University of Kwa-Zulu Natal, Private Bag X54001, Durban, 4000 South Africa; 3 BeauSci Ltd., Waterbeach, Cambridge, CB25 9TL UK; 4 Agronomy Department, University of Florida, Gainesville, FL USA; 5 Center for Genomics and Biotechnology, Haixia Institute of Science and Technology, Fujian Agriculture and Forestry University, Fuzhou, China; 6 Plant Molecular and Biology Program, Genetics Institute, University of Florida, Gainesville, FL USA

**Keywords:** *Tripidium*, Saccharinae, *saccharum* complex, Plastids, Phylogenetics, Taxonomy, Sorghinae, Assembly, Gene loci

## Abstract

**Background:**

For over 50 years, attempts have been made to introgress agronomically useful traits from *Erianthus* sect. *Ripidium* (*Tripidium*) species into sugarcane based on both genera being part of the ‘Saccharum Complex’, an interbreeding group of species believed to be involved in the origins of sugarcane. However, recent low copy number gene studies indicate that *Tripidium* and *Saccharum* are more divergent than previously thought. The extent of genus *Tripidium* has not been fully explored and many species that should be included in *Tripidium* are still classified as *Saccharum*. Moreover, *Tripidium* is currently defined as *incertae sedis* within the Andropogoneae, though it has been suggested that members of this genus are related to the Germainiinae.

**Results:**

Eight newly-sequenced chloroplasts from potential *Tripidium* species were combined in a phylogenetic study with 46 members of the Panicoideae, including seven *Saccharum* accessions, two *Miscanthidium* and three *Miscanthus* species. A robust chloroplast phylogeny was generated and comparison with a gene locus phylogeny clearly places a monophyletic *Tripidium* clade outside the bounds of the Saccharinae. A key to the currently identified *Tripidium* species is presented.

**Conclusion:**

For the first time, we have undertaken a large-scale whole plastid study of eight newly assembled *Tripidium* accessions and a gene locus study of five *Tripidium* accessions. Our findings show that *Tripidium* and *Saccharum* are 8 million years divergent, last sharing a common ancestor 12 million years ago. We demonstrate that four species should be removed from *Saccharum*/*Erianthus* and included in genus *Tripidium*. In a genome context, we show that *Tripidium* evolved from a common ancestor with and extended Germainiinae clade formed from *Germainia*, *Eriochrysis*, *Apocopis*, *Pogonatherum* and *Imperata*. We re-define the ‘Saccharum complex’ to a group of genera that can interbreed in the wild and extend the Saccharinae to include *Sarga* along with *Sorghastrum, Microstegium vimineum and Polytrias* (but excluding *Sorghum*). Monophyly of genus *Tripidium* is confirmed and the genus is expanded to include *Tripidium arundinaceum*, *Tripidium procerum*, *Tripidium kanashiroi* and *Tripidium rufipilum.* As a consequence, these species are excluded from genus *Saccharum*. Moreover, we demonstrate that genus *Tripidium* is distinct from the Germainiinae.

**Electronic supplementary material:**

The online version of this article (10.1186/s12862-019-1356-9) contains supplementary material, which is available to authorized users.

## Background

Sugarcane (complex hybrids of *Saccharum cultum* Lloyd Evans and Joshi, *Saccharum officinarum* L. and *Saccharum spontaneum* L. [1]) ranks amongst the top-10 crop species worldwide [2]. Sugarcane also provides between 60 and 70% of total world sugar output and is a major source of bioethanol [2]. However, sugarcane production is dependent on rich soil and plentiful water supply. Sugarcane growth is also highly temperature dependent, with an optimum of 27 °C and an optimal humidity of 85% [3]. This means that the sugarcane-growing belt is limited to the tropical and subtropical regions of the globe. As a result, sugarcane breeders have long had an interest in introgressing traits from sugarcane’s relatives to alter the growing range of sugarcane, to make the plant more productive, to increase its stress tolerance, and to make it more efficient in terms of resource usage (water, soil conditions, nitrogen) [4]. For optimal breeding success, particularly if using genera outside core *Saccharum* L., an accurate phylogenetic relationship between *Saccharum* and its purported relatives is a paramount necessity.

The concept of the ‘Saccharum complex’ as a set of interbreeding species and the Saccharinae as a subtribe of sugarcane’s close relatives has remained largely unchallenged for 60 years [5] and has been at the core of all molecular and traditional breeding approaches. These relationships can now be re-evaluated in the light of modern molecular taxonomic approaches.

Ever since Mukherjee included *Erianthus* Michx. sect. *Ripidium* Henrard within the ‘Saccharum complex’ [5], a group of potentially interbreeding genera believed to be involved in the origins of modern sugarcane hybrids (*Saccharum* ×*officinarum*/*Saccharum* ×*cultum* [1]), considerable effort has been expended in introgressing *Erianthus* species into the sugarcane gene-pool.

Clayton and Renvoize [6] included *Erianthus* in the subgenus Saccharinae Griseb. (along with the genera *Eriochrysis* P. Beauv., *Eulalia* Knuth, *Eulaliopsis* Honda, *Homozeugos* Stapf, *Imperata* Crillo, *Lophopogon* Hack, *Microstegium* Nees, *Miscanthus* Andersson, *Pogonatherum* P. Beauv., *Polliniopsis* Hayata, *Polytrias* Hack, *Saccharum* and *Spodiopogon* Trin), suggesting that *Erianthus* was closely allied to *Saccharum*. Indeed, this paper has had considerable taxonomic influence and Kew’s GrassBase defines former *Erianthus* species as part of the *Saccharum* genus [7].

However, the paper of Hodkinson et al. [8], though using relatively few characters for their phylogeny, demonstrated that *Erianthus* was not monophyletic. The Old World *Erianthus* species (sect. *Ripidium*) clustered as sister to *Eulalia* and *Zea* L., whilst the New World species and *Erianthus rockii* Keng (*Saccharum longisetosum* (Andersson) V. Naray.) clustered as sister to *Miscanthidium* Stapf. Hodkinson et al. [8] proposed the name *Ripidium* (following Grassl [9] and von Trinius [10]) for the genus corresponding to Old World *Erianthus* species. This name, however, was found to be invalid, as Berhnardi [11] had already proposed *Ripidium* for a fern species within the *Schizaeaceae*, and Valdés and Scholz [12] proposed *Tripidium* as a replacement.

To fully understand the problems associated with the naming and taxonomic positioning of *Erianthus*/*Tripidium* species, we must be fully conversant with the taxonomic origins and derivations of these genera and the potential use of species within them to sugarcane breeders.

The Old World species of *Erianthus* are highly caespitose (forming dense clumps); indeed, Grassl [9] defined them as ‘tufted bunch grass types’ and it was this dense bunching habit and efficient ratooning that initially led sugarcane breeders to attempt the introgression of *Erianthus* characteristics into sugarcane. The deep rhizomes of *Erianthus* species also mean that they have improved water use and nutrient use efficiency as compared with members of genus *Saccharum* (even some highly rhizomatous *Saccharum spontaneum* accessions) [13].

Genus *Erianthus* was first defined by Michaux [14], with the name being derived from the Greek ‘*Erion*’ (wool) and ‘*anthos*’ (flower), referring to the woolly glumes possessed by members of the genus. The New World species are found in the Americas, whilst the Old World Species occur from Mediterranean Europe through India, China, South East Asia, New Guinea and Taiwan. As the Old World species (originally placed under the section *Ripidium*) have always been believed to be closest to *Saccharum* (and part of the ‘Saccharum complex’ and the Saccharinae) they are analysed in detail within this paper. For reference, the nomenclatural history of species within *Erianthus* sect *Ripidium* (*Tripidium*) is given in Table 1.

**Table 1:** History of the nomenclature of species within *Erianthus* sect *Ripidium* (*Tripidium*)

Basionym	Commonly used name	Grassl re-classification [9]	Valdés et al. Re-classification [12]	GrassBase definition [7]	Tropicos definition [47]	Notes
*Saccharum arundinaceum* Retz.	*E. arundinaceus* (Retz) Jesw	*Ripidium arundinaceum*		*Saccharum arundinaceum* Retz.	*Saccharum arundinaceum* Retz.	
Erianthus kanashiroi Ohwi.	*E. kanashiroi* Ohwi	*Ripidium kanashiroi*		Saccharum kanashiroi Ohwi	*Saccharum kanashiroi* Ohwi	
*Saccharum procerum* Roxb.	*E. procerus* (Roxb.)	*Ripidium procerum*		*Saccharum procerum* Roxb.	*Saccharum procerum* Roxb.	
*Andropogon ravennae* L.	*E. ravennae* (L.) P. Beauv. (syn *R. ravennae* (L.) Trin.)		*Tripidium ravennae* (L.) Scholz	*Saccharum ravennae* (L.) L.	*Tripidium ravennae* (L.) H. Scholz	
*E. elephantinus* Hook. F.	*E. elephantinus* Hook. F.	*Ripidium elephantinum*				Synonymous with *S.ravennae* [85]
*Saccharum bengalense* Retz.	*E. bengalense* (Retz.)	*Ripidium bengalense*	*Tripidium bengalense* (Retz) Scholz	*Saccharum bengalense* Retz.	*Tripidium bengalense*	
*Andropogon strictus* Host	*E. strictus* (Host) Borbá (syn. *R. strictum* (Host) Trin.)		*Tripidium strictum* (Host) Scholz	*Saccharum strictum* (Host) Spreng.	*Saccharum strictum* (Host) Spreng.	
	*E. hostii* Griseb					Synonymous with *S. strictum* [86]
*Erianthus rufipilus* (Steud.) Griseb.	*Erianthus rufipilus* (Steud.) Griseb.	*Miscanthus rufipilus*		*Saccharum rufipilum* Steud.	*Saccharum rufipilum* Steud.	
	*E. longesetosus* Anderss	*Eccolipus longesetosus*				Synonymous with *Saccharum strictum* [87]
	*E. hookeri Hack*	*Eccolipus hookeri*				Synonymous with *Saccharum strictum* [87]
*Eulalia japonica* Trin.	*Ripidium japonicum (Trin.) Trin.;*					Synonymous with *Miscanthus floridulus* (Labill.) Warb. ex K. Schum. & Lauterb. [85]

The above table gives a history of the nomenclature of species within *Erianthus* sect *Ripidium* (*Tripidium*) starting with the Basionym (original name), followed by the treatment of Grassl [9], the re-classification of Valdés et al. [12], the current definition in Tropicos [47] and the current definition in GrassBase [7]. The notes column denotes species that have been re-classified as being synonymous with previously defined species. All species in the table are from the Old World

Following the work of Valdés et al. [12], genus *Tripidium* currently contains three accepted members: *Tripidium ravennae* (L.) H. Scholz, *Tripidium bengalense* (Retz) H. Scholz and *Tripidium strictum* (Host) H. Scholz. NCBI’s taxonomy also places all species detailed in Table 1 within genus *Saccharum*, apart from *Tripidium ravennae*, which is defined as the type species for *Tripidium*, and *Tripidium bengalense* [15]. This leaves four additional species (*Saccharum rufipilum*, *Saccharum procerum*, *Saccharum arundinaceum* and *Saccharum kanashiroi*) that could be members of genus *Tripidium*, all of which were analysed in this study.

A recent publication by Welker et al. [16], examining five low copy number gene loci, placed the Old World *Tripidium* (*Erianthus*) species as sister to a clade formed by the core Andropogoneae, the core Saccharinae and the core Sorghinae (though with relatively poor support). The New World species (*Saccharum giganteum* (Walter) Pers. [*Erianthus giganteus* (Walter) P. Beauv.], *Saccharum asperum* (Nees) Steud. [*Erianthus asper* Nees], *Saccharum angustifolium* (Nees) Trin. [*Erianthus angustifolius* Nees], and *Saccharum villosum* Steud [*Erianthus trinii* (Hack.) Hack.]) were placed within the *Miscanthus*/*Saccharum* (Saccharinae *s.s.*) clade. However, the positioning of *Tripidium* within the phylogeny of Welker et al. [16] differed slightly from that of the earlier paper of Estep et al. [17]. These authors did, however, propose the recognition of genus *Tripidium* as separate from *Saccharum*. Though they included both *Erianthus arundinaceus* and *Erianthus* (*Tripidium*) *ravennae*, there was no large-scale treatment of *Tripidium* species and no dating information on the divergence of *Tripidium* in their study. The paper of Soreng et al. [18] attempting to classify the Poaceae as whole, defined *Tripidium* as *incertae sedis*, but they did exclude the genus from *Saccharum* and suggested that *Tripidium* could be allied to the Germainiinae (*Germainia* and related genera). This uncertain placement stems from the reorganization of the Saccharinae and Germainiinae, which left four genera: *Eriochrysis*, *Imperata*, *Pogonatherum* and *Tripidium*, as *incertae sedis* [18]. Thus, whilst there is some agreement that genus *Tripidium* exists, there is no consensus on this finding, and a full circumscription of *Tripidium* has not been undertaken. Moreover, the species most commonly used in sugarcane introgression breeding (*Erianthus arundinaceus*) has never formally been included in genus *Tripidium*.

A larger-scale phylogenetic study with more extensive sampling of complete plastomes and genomic loci was always needed before a recommendation could be proposed for the confident circumscription and placement of *Tripidium* or any of the other genera. All four of the Andropogoneae genera currently defined as *incertae sedis* are included in the present study, with a large-scale sampling of *Tripidium* species. It is especially important to compare across genomic and plastome datasets, particularly as the role of hybridization in driving evolution, most especially in plant species, has recently received resurgent attention [19, 20]. Indeed, molecular studies from a variety of taxa across the tree of life have increasingly acknowledged that hybridization is an important source of evolutionary novelty [21, 22]. Pirie et al. [23] revealed ancient reticulation in the *Danthonioideae* (Poaceae) and ancient reticulation has been revealed in a range of plant genera [19, 24–28]. This is particularly important where there have been ancient rapid radiations [26], such as in the Poaceae [17]. As a result, the comparison of genome-based and plastome-based datasets is needed to examine the possibility of reticulate evolution within a genus, particularly in grasses where hybridization between lineages is especially common [29, 30].

Based on the existing evidence, it can be concluded that genus *Erianthus* is not monophyletic and that the New World *Erianthus* species are separate from the Old World species. Whilst the New World species may be allied to the *Saccharum* genus, the Old World species are not. Thus, the current inclusion of all former *Erianthus* species into *Saccharum* (as at Kew’s GrassBase [7] and the NCBI Taxonomy [31]) is almost certainly erroneous and is in dire need of re-evaluation.

The *Tripidium* (*Erianthus* sect. *Ripidium*) genus includes members with chromosome numbers of 2n = 10, 20, 30, 40 and 60 [12]. Though, in common with other members of the Panicoideae, the base number is x = 10, which is the same as for genus *Saccharum* [32]. As a genus, *Tripidium* possesses a number of agronomically useful phenotypic features namely: cold tolerance, drought tolerance, heat tolerance, salt tolerance, disease resistance, improved vigour, dense culm spacing and increased ratooning [13]. As *Erianthus* was included in the ‘Saccharum complex’ [5, 33] sugarcane breeders have long attempted to introgress the agronomic properties of *Erianthus* into sugarcane. Typically, introgression breeding involves *Erianthus arundinaceus* (*Tripidium arundinaceum*), but it has been difficult to both generate fertile progeny and to identify true hybrids [34]. Subsequent experiments have attempted to utilize cytogenetic tools such as GISH and species-specific markers to follow DNA transmission so that true hybrids can be identified at the seedling stage; and to follow *Erianthus* genome regions into subsequent progeny [35–38]. GISH [35] revealed that chromosome elimination occurs in *Saccharum* hybrid × *E. arundinaceus* hybrids, indicating that there might be a greater evolutionary distance between *Saccharum* and *Erianthus* than predicted solely on the basis of morphological characteristics. Recent studies have shown that the small percentage of true hybrids obtained in *Saccharum* hybrid × *E. arundinaceus* crossings are highly aneuploid, with loss and duplication of *E. arundinaceus* chromosomes as well as frequent interspecific recombinations between sugarcane and *E. arundinaceus* chromosomes [4, 39]. This phenomenon is characteristic of intergeneric hybridizations (wide crosses between evolutionarily very divergent plant species), as exemplified by oat/maize partial hybrids [40].

As considerable effort has been placed in introgressing *Erianthus* into sugarcane (typically with poor success) the question of the evolutionary distance between the two genera has both economic and taxonomic relevance. Particularly as molecular data are casting significant doubts on the veracity of the ‘Saccharum complex’ as a whole [41]. Clarifying the taxonomic position of the Old World *Erianthus* species (the most commonly used in introgression breeding) within the Andropogoneae and to the *Saccharum* *sensu stricto* species will help drive forward knowledge-based breeding in sugarcane.

We have sequenced and assembled complete chloroplasts from six *Tripidium* accessions from the South African Sugarcane Research Institute (two of known species and three re-classified as part of this study) and two accessions of known species from the USDA collection as well as an additional *Saccharum spontaneum* accession [42]. Our assemblies were integrated with 45 previously assembled chloroplasts genomes to yield the most comprehensive phylogenomic study of *Tripidium* and allied genera within the Andropogoneae thus far conducted. Moreover, a parallel analysis of five low copy number gene loci (63 species) was also performed.

Comparisons of whole chloroplast and gene loci phylogenies revealed that the *Tripidium* accessions were monophyletic, and sister to a clade formed by the core Andropogoneae and Saccharinae. They diverged from *Saccharum* at least 11 million years ago and are distal to the core Andropogoneae. We also reveal signals of reticulate evolution in genera that are currently defined as *incertae sedis* within the Andropogoneae.

This means that *Tripidium* should be the preferred genus name for these accessions and that they cannot be part of either the Saccharinae subtribe or the ‘Saccharum complex’. Moreover, *Tripidium* is circumscribed, based on both whole plastome and low copy number gene locus phylogenies, as a monophyletic grouping with seven confirmed species that is over 8 million years divergent from sugarcane. In addition, we show that the genera *Eriochrysis* and *Eulaliopsis* should also be excluded from the Saccharinae, as should *Sorghum* (based on low copy number gene phylogenetics). Genera *Saccharum*, *Miscanthidium*, *Miscanthus*, *Sarga*, and *Erianthus* do form a monophyletic group that could be termed the ‘Saccharinae’. However, of these, only *Miscanthus, Miscanthidium* and *Erianthus* are sufficiently evolutionarily close to *Saccharum* to allow for hybridization in the wild [1]. Thus, the ‘Saccharum complex’ as a group of interbreeding species should be limited to *Saccharum*, *Miscanthus*, *Miscanthidium* and the New World *Erianthus* species.

## Results

### Primer design

**Fig. 1:**
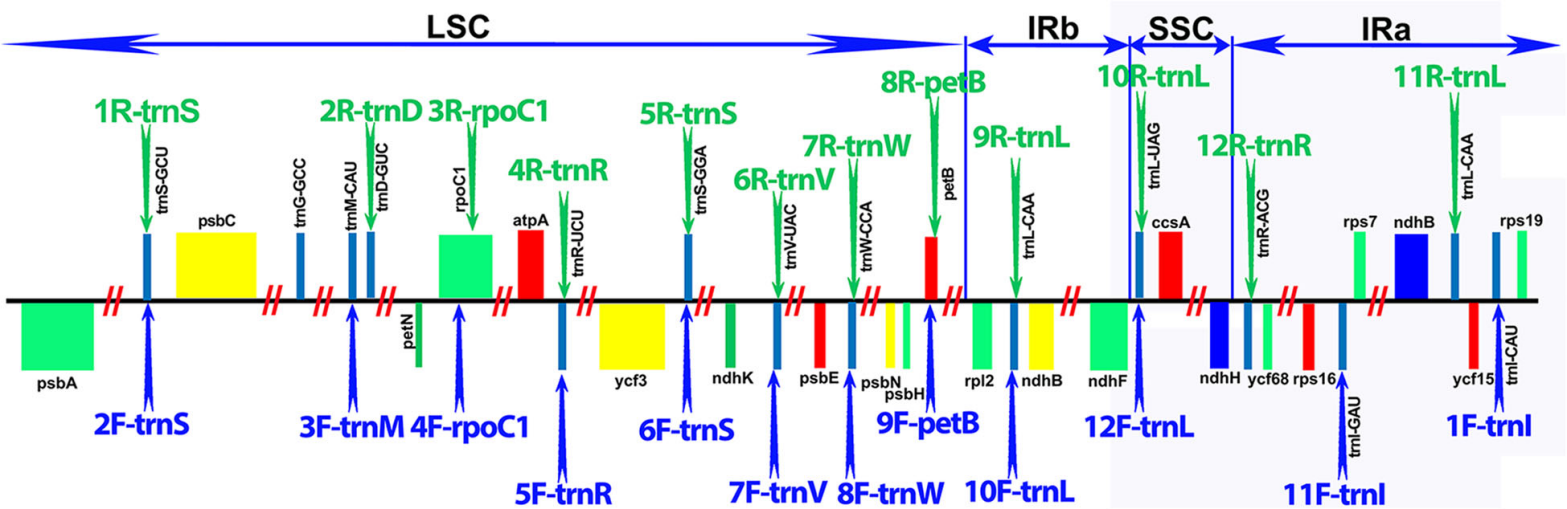
Mapping primer sets to a schematic of the *Saccharum arundinaceum* (*Erianthus arundinaceus*) chloroplast genome. Image shows a schematic of the *Erianthus arundinaceus* chloroplast genome, showing key genes and the locations of the four sections of the genome. Twelve of the primers designed to give full coverage of the genome are shown, with forward primers on the bottom and reverse primers on top

**Fig. 2:**
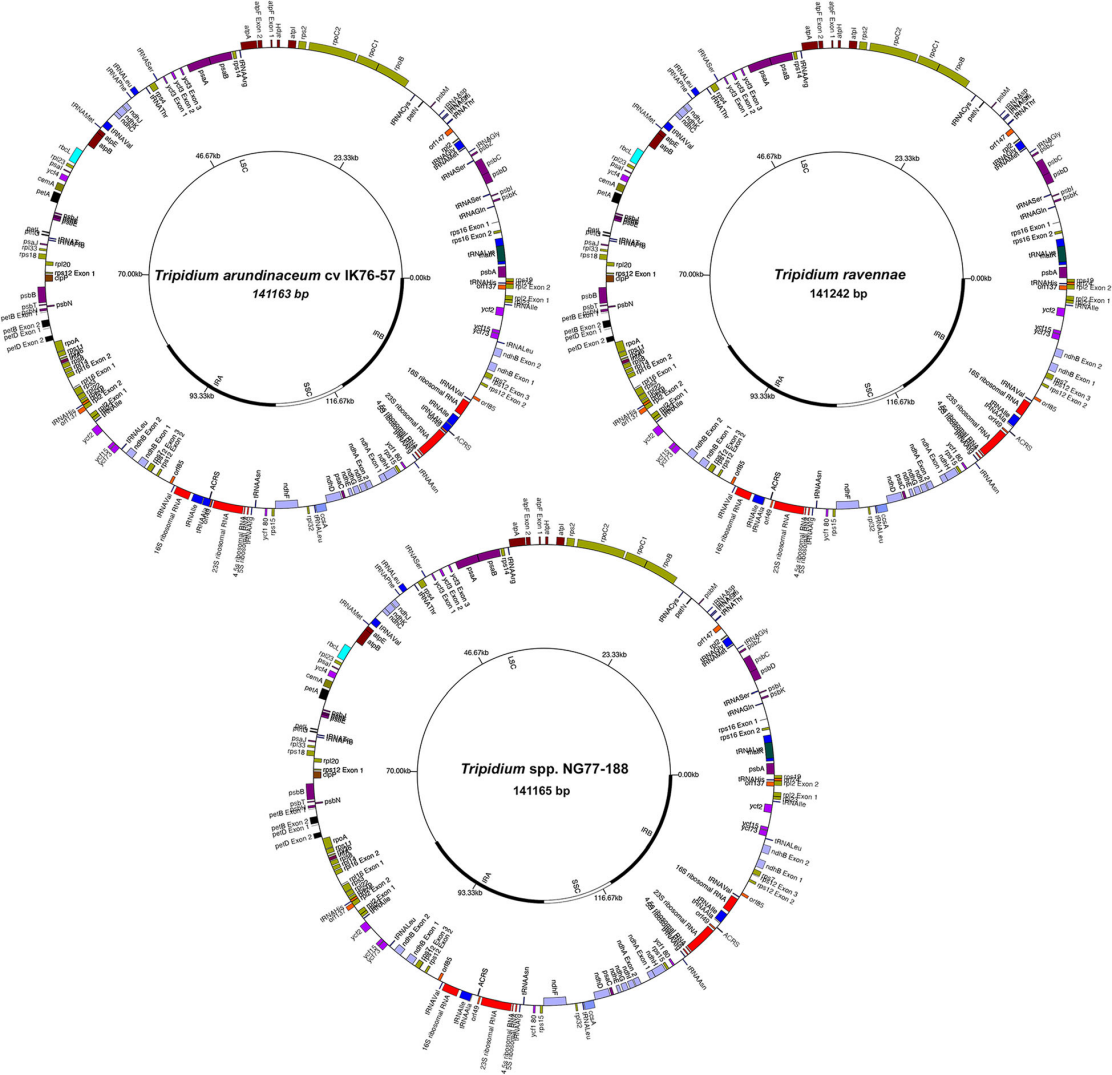
Schematic images for three *Tripidium* chloroplast assemblies. Shown are three schematic drawings for three chloroplast genomes assembled and annotated in the present study. Each image is characteristic of the three main *Tripidium* clades shown in Figs. 3 and 4. Protein coding genes are shown on the outer track, with forward strand genes on the outside and reverse strand genes on the inside. The inner track shows the extent of the large single copy region (LSC), small single copy region (SSC) and the two inverted repeats (IR_A_ and IR_B_). The first image, top left, represents the chloroplast of *Tripidium arundinaceum* cv IK76–57. The second image, top right, represents the chloroplast of *Tripidium ravennae*. The bottom image shows the chloroplast of *Tripidium* sp. NG77–188. Images were drawn with GenomeVx [88] prior to finishing with Adobe Illustrator

Chloroplast amplification primers (an additional PDF file details these Additional file 1) were designed to be universal to the Panicoideae, using *Saccharum* hybrid, *Miscanthus sinensis*, *Zea mays* and *Cenchrus americanus* plastids as references. Wherever possible, tRNAs were used as sites of primer design (these tend to be evolutionarily well conserved). The majority of tRNA-based primers were designed manually with a target melting temperature of 78 °C. For optimal PCR, amplicons were limited to a maximum 20,000 bases. In the few cases where there were no tRNAs available to design primers, the region was input into NCBI’s primer design tool [43]. Final primers were checked with Amplify4 [44] for specificity, primer dimerization and universality. Figure 1 shows a schematic mapping of predicted and subsequently assembled amplicons to the published *E. arundinaceus* chloroplast genome. There are two gaps in the map, within the IR_B_ region. However, as these two gaps are identical to the sequence on the IR_A_ inverted repeat, we still have complete coverage of the chloroplast (Fig. 2). Primer pair 13 is not shown on the schematic, as this was designed solely to assist with assembly because SPAdes (our assembler of choice) is sensitive to large troughs and peaks in the assembly graph. As the inverted repeats have double the coverage as compared to the SSC (short single copy) region separating them, an additional primer pair covering the core of the SSC region was designed to increase and even out read coverage in this region. Gel images for the amplicons generated for the *Tripidium* species prior to sequencing are shown as an additional PDF file (Additional file 2).

### Phenotypic analyses

Formal phenotypic analyses were performed on the South African Sugarcane Research Institute’s collection of *Saccharum robustum*, *Saccharum officinarum*, *Saccharum spontaneum* and *Erianthus* collections (excluding probable hybrids). Based on the possession of ciliate auricles and eciliate ligules, deep (rather than lateral) rhizomes, leaf sheaths that are longer than internode to internode distances, a conspicuous midrib on the leaf underside, leaf blades that taper towards both the tip and sheath and caespitose (clump-forming) natures six accessions were re-classified as Old World *Erianthus* types which probably belonged to genus *Tripidium* (see Kew’s GrassBase for detailed species descriptions, which were strictly adhered to [45]). Accessions showing these features were phenotypically typed in detail and the following accessions were re-classified based on this study: *Saccharum spontaneum* cv Spontaneum 1 to *Tripidium arundinaceum* cv SA-E1; *Saccharum spontaneum* cv Spontaneum 2 to *Tripidium kanashiroi* cv SA-E2; *Saccharum robustum* cv IK76–417 to *Tripidium arundinaceum* cv IK76–417 and *Saccharum robustum* cv NG77–188 to *Tripidium* sp. cv NG77–188. Of these, *Tripidium* sp. cv NG77–188 was the most phenotypically divergent, and despite being clearly caespitose it was much shorter in stature with thin culms that had waxy bands both above and below the internode and narrow leaves. These accessions, along with one accession known to be *Erianthus arundinaceus,* and one known to be *Tripidium ravennae,* were subject to chloroplast amplification and sequencing.

Based on these studies, and additional phenotypic examination of all species, a new key to the identification of *Tripidium* species was prepared (Table 2). This is the first key to the identification of all *Tripidium* species.

**Table 2:** Key to the identification of *Tripidium* species

1a. Habitat: Perennial, caespitose	
2a. Glumes similar	
3a. Leaf blade surface smooth on both sides	
4a. Spikelet callus hairs white, 1.5× length of spikelets	
5a. Rhachis internode 3.5–4 mm long	
*Tripidium arundinaceum*	
5b. Rhachis internodes 6–7 mm long	
*Tripidium procerum*	
4b. Spikelet callus hairs yellow or red, 2–3× length of spikelet	
*Tripidium rufipilum*	
3b. Leaf blade surface scabrous — rough on both sides	
*Tripidium strictum*^‡^	
2b. Glumes dissimilar	
6a. Glumes exceeding apex of florets, lower glumes elliptic, upper glumes lanceolate	
*Tripidium ravennae*^‡^	
6b. Glumes 1× length of spikelet, lower glume 2 keeled, lower glume hairs 4–9 mm long, upper glume without keels	
*Tripidium bengalense*^‡^	
1b. Habitat: Perennial, culms solitary	
Key characters: Culms erect, 70–100 cm long, 5–15 mm diameter, rhizomes short, leaf blade hairy at base. Spikelet callus hairs 3–6 mm long.	
*Tripidium kanashiroi*	

Presented here is a key to the identification of *Tripidium* species. This key covers all currently accepted *Tripidium* species as well as those species moved into genus *Tripidium* as a consequence of the present study. The characters chosen in this key allow for simple disambiguation amongst the species. The three species marked with a double dagger (‡) superscript are the currently accepted members of *Tripidium*. The four remaining species were added to *Tripidium* as a result of this study

### Plastome assemblies

The eight *Tripidium* plastomes assembled in this study were remarkably similar in terms of size, gene and genome structures. Plastome length varied from 141,105–141,242 bp (*Tripidium rufipilum* and *Tripidium ravennae*, respectively), with a mean of 141,168 bp, the LSC (large single copy region) being the most variable. The *Saccharum spontaneum* SES196 dataset assembled in this study was cognate with the SES205A and SES234B *Saccharum spontaneum* chloroplasts that we had published previously (Additional file 3). All plastome assemblies had 84 protein-coding genes, 33 non-protein coding genes (ribosomal RNA and tRNAs), three pseudogenes and four origins of replication (Fig. 2) [all counts are for unique genes and exclude duplicates on the second, IR_B_, inverted repeat].

Plastome sequences derived from PCR amplification (SASRI data) had average read coverage of 330×, whilst plastome sequences derived from reduced representation whole genome amplification sequencing (USDA accessions) had an average of 67× coverage.

### Phylogenomic analyses

**Fig. 3:**
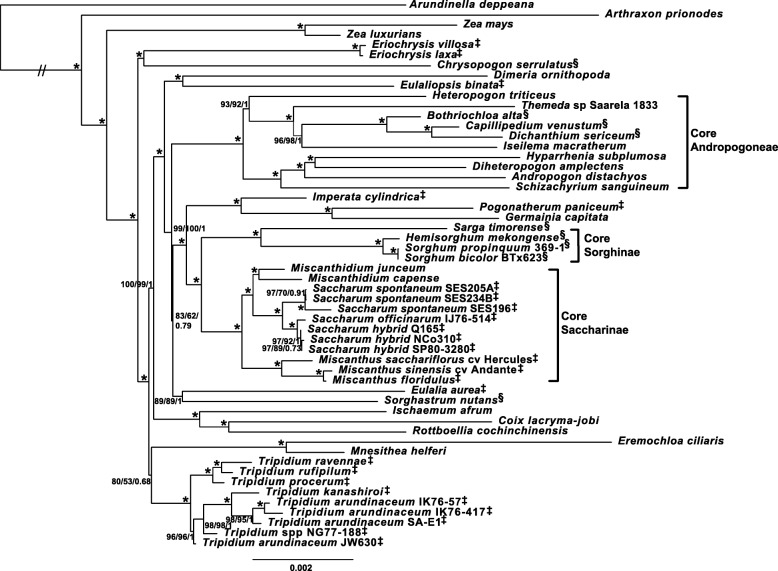
Whole plastid phylogram showing the relationships of *Tripidium* species to other members of the Andropogoneae. Phylogram, based on whole chloroplast alignment, representing the most likely topology from two independent runs of RAxML comparing whole chloroplast alignments of nine *Tripidium* accessions (eight sequenced and assembled in this study) with 45 representative members of the Andropogoneae (*Saccharum spontaneum* SES196 was also sequenced and assembled in this study). *Arundinella deppeana* is used as outgroup. The two slashes (//) indicate that the long branch linking the outgroup to the remainder of the tree has been reduced by 80% so that internal relationships can be more clearly seen. Numbers next to nodes represent SH-aLRT bootstrap, non-parametric bootstrap and Bayesian inference confidence values where * represents 100/100/1. The scale bar at the base of the phylogeny represents the expected number of substitutions per site. Brackets and annotation at the right of the phylogeny show the extent of the core Saccharinae, core Sorghinae and the core Andropogoneae. Images were drawn with FigTree [83] prior to finishing with Adobe Illustrator. Currently accepted members of the Saccharinae are represented by a double dagger (‡) and currently accepted members of the Sorghinae are represented by a section sign (§)

To test the possible influence of partitioning and the two inverted chloroplast repeats on the chloroplast-based phylogenies both maximum likelihood and Bayesian analyses were run with the second inverted repeat IR_B_ both present and absent from the alignment. Tree topologies in both cases were identical, and branch support showed less than 5% variance. However, backbone branch supports were relatively poor as were some internal branches, particularly within the *Tripidium* genus. Moreover, Neighbor-Joining, Parsimony Ratchet analyses and SH-aLRT analyses presented an alternate tree topology (particularly within *Tripidium* and for the relationship of *Sorghastrum* to the core Andropogoneae and Saccharinae). For an example topology (with supports) using the standard partitioning of the alignment into LSC, IR_A_ and SSC regions see the PDF document of the additional file (Additional file 4). As such, it was decided to further partition the LSC, IRA and SSC regions of the chloroplast into protein coding genes, RNA-coding genes and non-coding sequences (8 partitions in all) with all partitions analyzed independently. This is a much finer partitioning than is typically performed for whole chloroplast analyses and it yielded significantly improved branch supports and a more consistent tree topology. For the final partitioning scheme, two independent runs of RAxML (with different ‘-p’ seed numbers and 100 replicates) yielded the same tree topology, with the best log likelihood -lnL = 409,657.45 (Fig. 3). The current phylogeny focuses on the Andropogoneae, with *Arundinella deppeana* (a member of the Arundinelleae) as the outgroup.

Within the SASRI collection, accessions SA-E1 and SA-E2, were previously identified as *Saccharum spontaneum* and accessions NG77–188 and IK76–417 were previously identified as *Saccharum robustum*. This confusion of identification undoubtedly arose due to the presence of rhizomes and the clump-forming nature of these accessions. However, careful phenotypic analyses revealed that these accessions should more properly be included within genus *Tripidium*, a conclusion that is supported by their monophyly within our phylogeny, particularly if one follows Grassl [9] in excluding the ‘bunch grass’ types from *Saccharum*.

**Fig. 4:**
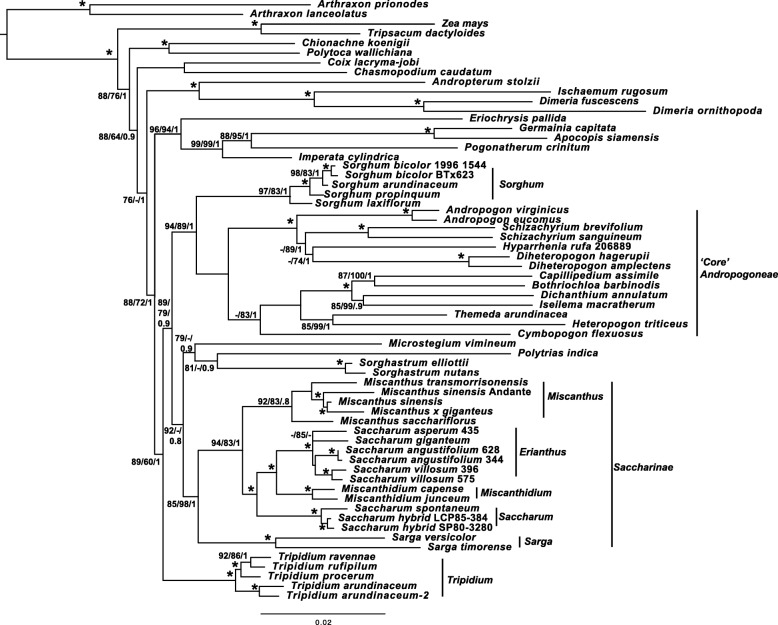
Phylogram based on five low copy number gene loci, depicting the relationships of *Tripidium* species and the Saccharinae to other members of the Andropogoneae. Phylogram, based on the concatenated alignments of five low copy number gene loci representing the most likely topology from two independent runs of RAxML comparing whole chloroplast alignments of six *Tripidium* accessions (three sequenced and assembled in this study) with 59 representative members of the Andropogoneae, but focussed on the Saccharinae and Sorghinae. *Arthraxon lanceolatus and Arthraxon prionodes* were employed as an outgroup. Numbers next to nodes represent SH-aLRT support, non-parametric bootstrap and Bayesian inference confidence values where * represents 100/100/1 and – represent a value below the cutoff (85% for SH-aLRT, 75% for non-parametric bootstrap and 0.7 for Bayesian Inference). The scale bar at the base of the phylogeny represents the expected number of substitutions per site. Vertical bars and annotation at the right of the phylogeny show the extent of the genera: *Sorghum*, *Miscanthus*, *Miscanthidium*, *Erianthus*, *Saccharum*, *Sarga* and *Tripidium* as well as the Saccharinae and core Andropogoneae. Images were drawn with FigTree [83] prior to finishing with Adobe Illustrator

Our gene locus phylogeny (Fig. 4) is based on the work of Estep et al. [17]. As in previous studies, terminal branches have good support, but internal nodes tend to have poorer bootstrap support (but good BI support), an indication of rapid radiation [17]. The topology is almost identical to the phylogeny presented by Welker et al. [16], with the exception that we place *Polytrias indica* as sister to *Sorghastrum* rather than sister to *Sorghum*. However, our placement of *Polytrias* agrees with the phylogeny of Estep et al. [17]. In addition, comparisons between the chloroplast (Fig. 3) and gene locus (Fig. 4) phylogenies show that both phylograms are largely congruent.

**Fig. 5:**
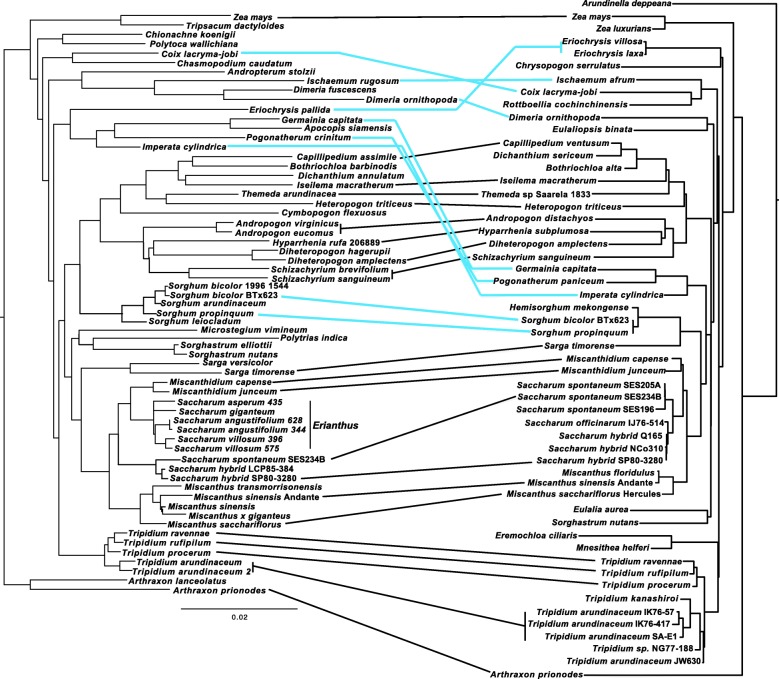
Tanglegram comparing the low copy number gene loci phylogeny and whole plastid phylogeny generated in this study. Direct comparison of the low copy number gene phylogeny (left) and the whole plastid phylogeny (right) generated in this study. To maximize congruence between the two phylograms, nodes were swapped with FigTree [83] prior to image merging in Adobe Illustrator. Black lines represent genera in cognate positions, whilst blue lines represent genera in discordant positions between the two phylogenies

A side-by-side comparison, presented as a tanglegram (Fig. 5) shows that the gene locus phylogeny and the whole chloroplast genome phylogeny are largely congruent, with only eight genera differing between them. The major differences being the relative positioning of the core *Sorghinae*, a monophyletic clade formed from *Eriochrysis*, *Imperata*, *Pogonatherum*, *Apocopis* and *Germania* and a monophyletic clade formed from *Ischaemum*, *Coix* and *Rottboellia* in the chloroplast phylogeny which splits into two more distal clades in the gene locus phylogeny. However, monophyly of *Coix, Chasmopodium* and *Rottboellia* is confirmed by the work of Estep et al. [17].

*Imperata*, *Pogonatherum* and *Germainia* remain monophyletic in both phylogenies, but this clade forms an outgroup to the core Andropogoneae and Saccharinae, with good support, in the chloroplast phylogeny (Figs. 3 and 5) whilst it is sister to *Eriochrysis* with this entire clade being an outgroup to *Tripidium* in the gene locus phylogeny. The core Sorghinae form a monophyletic grouping in both phylogenies, but are placed as sister to *Sarga* in the chloroplast phylogeny and as sister to the core Andropogoneae in the gene locus phylogeny.

*Coix*, *Ischaemum* and *Rottboellia* form a monophyletic grouping in the chloroplast phylogeny but monophyly of this group is not supported by the gene locus phylogeny (Figs. 4 and 5) where *Coix* is sister to *Chasmopodium* and this grouping is immediately antecedent to a clade formed by *Andropterum*, *Ischaemum* and *Dimeria*.

Both phylogenies (Fig. 5) support *Tripidium* as a monophyletic grouping which, in the chloroplast phylogeny (Fig. 3), has *Eremochloa ciliaris* and *Mnesithea helferi* as a sister clade (with relatively weak support). Our two phylogenies support the division of *Tripidium* into a *Tripidium arundinaceum/kanashiroi* clade and a *T. procerum/T. rufipilum and T. ravennae* clade, with near 100% branch support in both groups.

Within the core *Andropogoneae*, a division into a clade represented by *Andropogon* and a clade represented by *Bothriochloa* is supported by both phylogenies. Both nuclear locus and plastome phylogenies also support *Sarga* as being sister to the core Saccharinae, which is represented by *Miscanthus, Miscanthidium* and *Saccharum*. The gene locus phylogeny (Fig. 4) places New World Erianthus species (represented by the type species, *Erianthus* (*Saccharum*) *giganteus* as sister to *Miscanthidium*. All these genera receive 100% support in both phylogenies as being monophyletic.

**Fig. 6:**
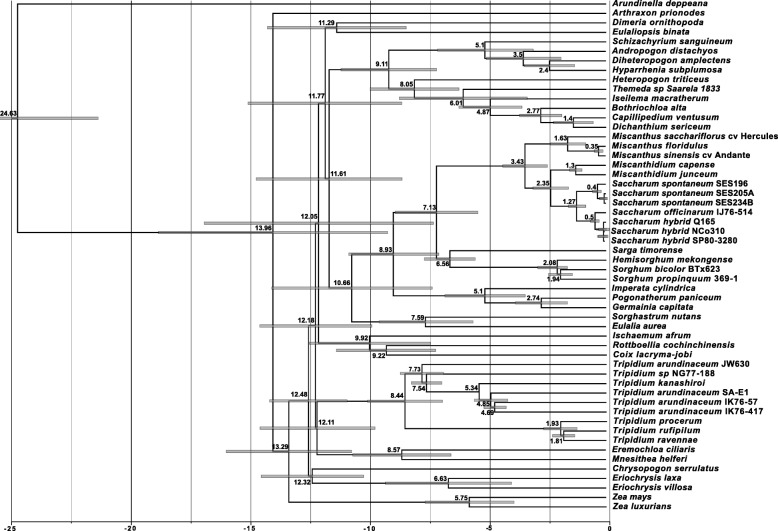
Chronogram showing the evolutionary distances (in millions of years) between the species and accessions analysed in chloroplast phylogenetic analysis of this study. Numbers to the left of nodes show the age of the node in millions of years ago (Mya). Node bars give the 95% highest posterior density (HPD) for the node height. The scale at the bottom gives millions of years before present in 2.5 million year increments. *Arundinella deppeana* is employed as the outgroup. Images were drawn with FigTree [83] prior to finishing with Adobe Illustrator

**Fig. 7:**
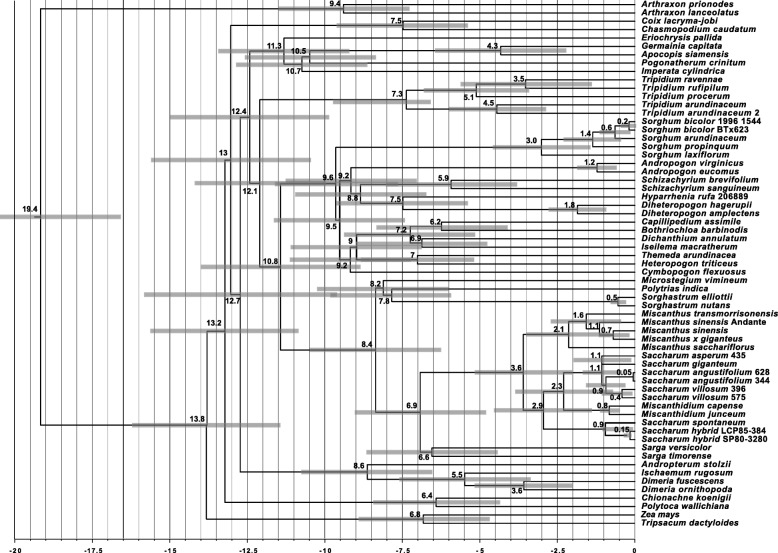
Chronogram showing the evolutionary distances (in millions of years) between the species and accessions analysed in the low copy gene locus phylogenetic analysis of this study. Numbers to the left of nodes show the age of the node in millions of years ago (Mya). Node bars give the 95% highest posterior density (HPD) for the node height. The scale at the bottom gives millions of years before present in 2.5 million year increments. *Arthraxon lanceolatus and Arthraxon prionodes* are employed as an outgroup. Images were drawn with FigTree [83] prior to finishing with Adobe Illustrator

The chloroplast phylogeny chronogram (Fig. 6) and gene locus chronogram (Fig. 7) (both chronograms calibrated on the divergence of *Zea mays* at ~ 13.8 Mya) concur in placing the divergence of *Tripidium* at 12.1–12.2 million years ago, with the crown *Tripidium* species diverging at 7.3–8.4 million years ago (Fig. 5).

Core Andropogoneae diverged from the Saccharinae at 10.8–11.6 million years ago. The chloroplast-based chronogram (Fig. 6) gives an age for the emergence of the Andropogoneae with *Arundinella deppeana* at 24.6 Mya. This is consistent with, but more ancient than, the work of Vicentini et al. [46], placing the origins of *Arundinella* at 19 Mya. Our age of divergence of *Saccharum* and *Miscanthus* at 3.4–3.6 Mya is consistent with previous studies that placed this divergence at 3.8 Mya [1, 17]. The split between *Saccharum spontaneum* and the crown *Saccharum* species is consistent with our previous work at 0.9–1.3 Mya [1].

## Discussion

The concept of the ‘Saccharum Complex’ as a collection of interbreeding species resulting in the evolution of sugarcane has had a profound, and probably detrimental, effect not only on sugarcane breeding, but also on the taxonomy of the Andropogoneae as a whole. Many of these ‘Saccharum complex’ species have been placed wholesale into the subtribe ‘Saccharinae’. A good case in point is *Erianthus* sect. *Ripidium*. The original definition of this section recognized eight Old World *Erianthus* species, which Grassl [9] placed within genus *Ripidium*. Tropicos, following Scholz [12] places three species within genus *Tripidium* [47] though Kew’s GrassBase places all these species within *Saccharum* [7]. These inconsistencies, and the current definition of genus *Tripidium* as *incertae sedis* within the Andropogoneae necessitates a large-scale molecular phylogenetic study to verify the validity of the genus. Our data indicates that a more complex partitioning scheme than is typically used for phylogenomic studies (see Materials and Methods for details) yields improved branch support and improved topological robustness. As a result, this partitioning scheme was employed for all phylogenetic analyses reported in this paper. This finding of improved topological support with finer-grained partitioning is comparable to the study of Folk et al. [19], analysing *Heuchera*.

For the current study, complete chloroplast sequences of eight potential *Tripidium* accessions and a single *Saccharum spontaneum* accession as well as low copy number gene loci (apo1, d8, ep2 exon 7, ep2 exon8 and rep1) were assembled. During assembly and sequencing of the gene loci, we observed no secondary gene copies in *Tripidium*, indicating that there has been no recent hybridization within this genus. Our gene loci assemblies bolstered sampling in *Tripidium*, *Saccharum*, *Miscanthus* and *Sorghum* as compared with earlier studies [16, 17]. By integrating with previously published data [17, 48] we were able to generate a low copy number gene phylogeny (Fig. 4) to complement our whole chloroplast phylogeny (Fig. 3).

Whilst the chloroplast (Fig. 3) and nuclear gene locus (Fig. 4) phylogenies are broadly congruent, a close comparison, presented as a tanglegram (Fig. 5) demonstrates several points of large-scale phylogenomic discord where the chloroplast and nuclear signals cannot be reconciled. Discordance among genomes has long been observed in plants [49]. Both hybridization and incomplete lineage sampling (ILS) cause phylogenetic discordance, yet expected gene tree distributions under these processes are different.

Manually comparing the two phylogenies, all discordant branches have good support (greater than 85% SH-aLRT, 70% bootstrap and 0.9 BI), which means false identification of conflict could only occur in cases of systematic error [23]. Moreover, the majority of the examples of incongruence presented in Fig. 5 involve branches that are distant in the tree. Mechanistically, the pattern of discordance observed in Fig. 5 could be explained as resulting either from ancient hybridization or from deep lineage sorting (coalescent stochasticity). However, as hybridization is particularly common in grasses (see [29, 30]) ancient reticulation affords us the most parsimonious explanation for the discordance. This is especially the case for the Andropogoneae, where the chronograms (Figs. 6 and 7) show multiple lineages that diverged rapidly between 12.5 and 11.6 million years ago. This corresponds quite well with the central part of the Middle Miocene Climate Transition (MMCT) [50] and the start of the diversification of C4 grasses [51]. Rapid radiation can often prove problematic for phylogenetic analyses, particularly near the backbone of the phylogenetic tree [23], though our more comprehensive partitioning scheme does generally improve branch support in this region (Additional file 4 and Fig. 3).

Our findings are consistent with reticulate evolution being one of the driving forces behind this radiation. It is also perhaps unsurprising that three genera currently defined as *incertae sedis* within the Andropogoneae (*Eriochrysis*, *Imperata*, *Pogonatherum*) show signals of ancient reticulation with highly discordant genomic and plastid phylogenetic signals. Two of these genera, *Imperata* and *Pogonatherum* form a monophyletic grouping with *Germainia* in both chloroplast and genomic phylogenies (Figs. 3 and 4). The Germainiinae are currently defined as *Apocopis*, *Germainia* and *Trachypogon*. Though *Trachypogon* was not represented in our taxon sampling, *Apocopis* is represented in the low copy number gene locus phylogeny (Fig. 4) and is sister to *Germainia*. The surprise is that the gene locus phylogeny places *Eriochrysis* as the outgroup to the Germainiinae (a finding that is consistent with previous studies [16, 17]). It would seem that reticulation occurred early in the evolutionary history of the Germainiinae, so that genomic and plastid evolutionary histories differ. Based on the low copy number gene phylogeny (Fig. 4), our data support the inclusion of *Imperata* and *Pogonatherum* within the Germainiinae.

The evolutionary histories of *Coix*, *Dimeria* and *Ischaemum* differ greatly between the gene locus and chloroplast phylogenies (Fig. 5) and increased taxon sampling may be required to resolve the true reticulate origins of these species.

Possibly the most surprising discordance between the genomic and plastid phylogenies is the positioning of *Sorghum*. In the chloroplast phylogeny (Fig. 3) it emerges as sister to *Sarga*, with this joint clade being sister to the Saccharinae. However, in the genomic gene locus phylogeny, *Sorghum* is sister to the core Andropogoneae. This new placement of *Sorghum* is consistent with the work of Hawkins et al. [48] and our recent work on extended ITS sequences [52]. This means that *Sorghum* last shared a common ancestor with sugarcane 10.8 million years ago (Fig. 6) rather than the more commonly accepted 7.1 million years ago (Fig. 5). This also has implications for plastid/plastid+genomic datasets, which could lead to misleading phylogenetic interpretations.

Previous to this study, the complete plastid sequence of only a single *Erianthus* species; *Erianthus arundinaceus* has been published [53]. This is an East Asian accession (from the Shizuoka Prefecture of Japan). The SASRI *E. arundinaceus* accession (IK76–57) was collected in Indonesia (the IK designation represents a plant collected on the island of Kalimantan). The South African Sugarcane Research Institute accession SA-E1 was derived from India. Previous morphological studies [9] indicated that Indian and Indonesian accessions of *E. arundinaceus* were quite distinct, with Indian *E. arundinaceus* being closer to *E. ravennae* and *E. procerus*. Our phylogenies (Figs. 3, 4, and 5) show that genus *Tripidium* is monophyletic, with complete support. The genus is divided into two groupings, both with good support. However, *Tripidium arundinaceum* is clearly not monophyletic. The previously published Japanese accession forms an outgroup to our accession NG77–188, previously miss-identified as *Saccharum robustum*. These two species are basal to the remaining *Tripidium kanashiroi* and *Tripidium arundinaceum* accessions. *T. procerum*, *T. ravennae* and *T. rufipilum* cluster together and are sister to all the other *Tripidium* accessions. Indian *Tripidium arundinaceum* SA-E1 is basal to the crown *Tripidium arundinaceum* accessions, but is separate from *Tripidium* (*Erianthus*) *ravennae*. Thus, chloroplast data supports Indian *Tripidium arundinaceum* being phylogenetically more closely related to Indonesian *T. arundinaceum* than to *T. ravennae*.

Both chloroplast-based and low copy number gene locus based chronograms (Figs. 6 and 7) show that *Tripidium* diverged from the other grasses about 12.1 Mya. The two crown *Tripidium* clades diverged about 7.3 million years ago (Fig. 6). The chloroplast analysis (Fig. 3) places *Tripidium* sp. NG77–188 and *T. arundinaceum* JW630 as outgroups to the crown clade formed by *Tripidium kanashiroi* and the *Tripidium arundinaceum* species. Monophyly of this entire grouping has 100% support in both plastome and low copy number gene locus phylogenies (with identical topologies) and monophyly of the two sub-groupings within *Tripidium* has excellent support. Thus, our study provides two independent analyses supporting *Tripidium* as a monophyletic genus that last shared a common ancestor with *Saccharum* species 12.1 Mya. As such, we propose that *Tripidium* should be the preferred genus name for these species and that they should be removed from genus *Saccharum*.

Indeed, it is generally accepted that the demarcation line between species of Andropogoneae that could be members of the Saccharinae, and those which most certainly are not, are the core Andropogoneae. Species and genera that arose before the divergence of the core Andropogoneae (about 11.6 Mya) are not members of the Saccharinae, whilst genera that are sister to the core Andropogoneae could be members of the Saccharinae [54].

Our plastid phylogeny, (Fig. 3) shows that the crown *Tripidium* group formed by *T. kanashiroi* and three *T. arundinaceum* accessions have two basal accessions. The first of these is *T. arundinaceum* JW630, diverging 7.73 Mya (Fig. 5) and the second formed by *Tripidium* sp. NG77–188 diverging some 7.54 Mya. These are evolutionarily distinct from the crown *T. arundinaceum* group and separated from them by over 2 million years. *Tripidium* sp. NG77–188 is also morphologically distinct from the crown *T. arundinaceum* grouping. Despite being characterized as *T. arundinaceum*, accession JW630 is the most basal of the group and it is separated from the remaining *T. arundinaceum* accessions by another species, *T. kanashiroi*. The presence of *T. arundinaceum* accessions in the basal and crown groups means that *T. arundinaceum* is not monophyletic. Thus, *T. arundinaceum* JW630 is, undoubtedly, a different species from the core *T. arundinaceum* and *T. kanashiroi* species assembled in this study, and the naming and identification of these basal *Tripidium* species requires further investigation.

In our plastid analysis (Fig. 3), the monophyletic *Tripidium* clade is sister to a clade formed by *Eremochloa ciliaris* and *Mnesithea helferi.* It should be noted that Neighbor-Joining analysis supports the placement of *Eulalia* and *Sorghastrum*, but places *Mnesithea* and *Eulalia* as sister to *Tripidium*, but SH-aLRT supports the topology as presented. Indeed, support for *Eremochloa*/*Mnesithea* being sister to *Tripidium* is only slightly better than the topology where *Eremochloa*/*Mnesithea* is an immediate antecedent clade to *Tripidium*. This may be partly due to the rapid radiation seen in this portion of the phylogeny and increased sampling in this area may help resolve the uncertainty within the relative position of the *Eremochloa*/*Mnesithea* clade or uncertainty could be due to ancestral reticulate evolution. However, the uncertainty in the placement of these two clades does not affect our conclusions regarding *Tripidium*.

Our study therefore provides excellent support for *Tripidium* as a monophyletic genus that last shared a common ancestor with *Saccharum* species 12.2 Mya. As such, we propose that *Tripidium* should be the preferred genus name for these species and that they should be removed from genus *Saccharum*.

In addition, our recent study [1] gives a 3.4 million year window in which members of the Poaceae can hybridize in the wild. *Tripidium* species clearly fall outside this window and though they can be crossed by human mediation they cannot naturally hybridize in the wild. Indeed, our findings are entirely consistent with the problems in hybridizing these two very divergent genera that sugarcane breeders have encountered over the past 50 years. In addition, the large evolutionary distance between *Tripidium* and *Saccharum* explains why crosses between the genera result in the duplication and loss of incompatible chromosomes (as hybrids are intergeneric). Thus, in the strictest sense, those rare hybrids that occur between *Tripidium* species and *Saccharum* species should be defined as partial hybrids and not as true hybrids.

As only three species have previously been formally included in genus *Tripidium*, our findings show that the following new combinations are necessary: *Tripidium arundinaceum* (Retz.) Lloyd Evans comb. nov. based on *Saccharum arundinaceum* (Retz.); *Tripidium procerum* (Roxb.) Lloyd Evans comb. nov. based on *Saccharum procerum* (Roxb.); and *Tripidium kanashiroi* (Ohwi) Lloyd Evans comb. nov. based on *Saccharum kanashiroi* (Ohwi). We confirm by molecular phylogenetic data that *Saccharum rufipilum* is not a member of genus *Miscanthus* and is sister to *Tripidium ravennae*. As such, it must be brought into genus *Tripidium* as *Tripidium rufipilum* (Steud.) Lloyd Evans comb. nov. based on *Saccharum rufipilum* (Steud.). With the previous combinations, this results in *Tripidium* being a genus with seven confirmed species. To aid identification of these species, a new key to *Tripidium* identification is presented in Table 2. That Tripidium species’ physical characteristics can be ranked in this fashion is further evidence for the monophyly of *Tripidium* as a genus.

Our low copy number gene phylogeny places *Germainia* and the extended Germainiinae as a clade that is separate from *Tripidium*, but which is sister to *Tripidium*, the core Andropogoneae and the Saccharinae *sensu* *lato* as a whole. Thus *Tripidium* and *Germainia* are not closely allied as had previously been suggested [18].

As our low copy number gene locus phylogeny (Fig. 5) places *Sorghum* as an outgroup to the core Andropogoneae, we are left with a core Saccharinae subtribe of *Miscanthus*, *Miscanthidium* and *Saccharum*, which all lie within the wild crossing range of about 3.8 million years. Figure 3 clearly shows that the Saccharinae are not monophyletic and that three genera previously included in both the Saccharum complex and the Saccharinae (*Tripidium, Sorghum* and *Eriochrysis*) can be excluded as they are more distantly related to *Saccharum* than the core Andropogoneae. *Pogonatherum* and *Imperata* can also be excluded due to evolutionary distance, particularly if the low copy gene locus data (Fig. 4) is given precedence. It should be noted that genus *Miscanthidium* is not currently defined as part of the core Saccharinae (Fig. 3). Previously these species were included within *Miscanthus* [8] but our data clearly show that they are far closer to *Saccharum* than to *Miscanthus*. Our low copy number gene locus phylogeny (Fig. 4) also shows that New World *Erianthus* species are sister to *Miscanthidium*, thus they are part of the Saccharinae, but distinct from *Saccharum* (the *Erianthus* + *Miscanthidium* clade being sister to *Saccharum*).

Our phylogenies also demonstrate that the Sorghinae, as currently defined, is not monophyletic, with *Sorghastrum* (currently defined within the Sorghinae) clearly lying outside the Sorghinae, being sister to the core Saccharinae. Three other genera: *Bothriochloa*; *Capillipedium* and *Dichanthium* lie within the core Andropogoneae and can also be excluded from the Sorghinae. *Chrysopogon*, which is also currently included within the Sorghinae [6] can also be excluded as it is distal to the core Andropogoneae. The split between *Sorghum* and *Sarga* species is further emphasized as low copy number gene locus phylogenetics places *Sorghum* as sister to the core Andropogoneae, whilst *Sarga* is sister to the core Saccharinae.

Thus, our data supports a core Sorghinae clade formed only from *Hemisorghum* and *Sorghum* and a Saccharinae clade formed from the genera *Saccharum*, *Miscanthus, Miscanthidium* and *Erianthus* that has *Sarga* as a sister clade. Phylogenetically, we describe two new subtribes, as there is a natural clade formed from *Eriochrysis pallida*, *Germainia capitata*, *Apocopis siamensis*, *Pogonatherum crinitum* and *Imperata cylindrical* that can be encompassed within the Germainiinae. An extended clade formed from *Sarga, Miscanthus, Miscanthidium, Erianthus* and *Saccharum* could be given the subtribe name ‘Saccharinae’.

## Conclusion

The genera *Tripidium*, *Eulalia* and *Eriochrysis* should be excluded from the *Saccharinae* as they break the sub-tribe’s monophyly and are too distant from *Saccharum* to hybridize with *Saccharum* species in the wild. By the same argument *Chrysopogon, Bothriochloa*; *Capillipedium*, *Dichanthium* and *Sarga* should be excluded from the Sorghinae.

We also clearly demonstrate that genus *Tripidium* is not allied to Germainiiae, as suggested by Soreng et al. [18], though the Germainiiae are sister to *Tripidium*. Here we need to make a clear distinction between the ‘Saccharum complex’, a group of species that can interbreed in the wild (this can only include the genera: *Miscanthus*, *Miscanthidium* and *Saccharum*, as well as the New World *Erianthus* species [16]) and the Saccharinae, a taxonomically monophyletic grouping that includes genus *Saccharum* (in totality this subtribe would include *Saccharum*, *Miscanthus*, *Miscanthidium*, the New World *Erianthus* species and *Sarga* possibly with the inclusion of *Microstegium vimineum*, *Polytrias indica* and *Sorghastrum* which forms an outgroup to the ‘Saccharum complex’ (with good support)) based on low copy number gene phylogenetics (Fig. 4).

We also demonstrate, based on low copy gene locus phylogenetics, that genus *Erianthus*, as originally defined, is not monophyletic and should be divided into *Tripidium* and *Erianthus*, with genus *Erianthus* only including the New World species. Moreover *Erianthus* species are sister to *Miscanthidium* and are therefore phylogenetically distinct from *Saccharum*. As *Erianthus giganteus* is the type species we propose that New World *Erianthus* species should be removed from genus *Saccharum* and returned to genus *Erianthus*.

Despite over 50 years’ worth of efforts in introgression breeding, there has been little success in the generation of valid *Saccharum/Erianthus* hybrids. Our phylogeny clearly reveals that, as genera, *Saccharum* and *Tripidium* are 8.6 million years divergent, last sharing a common ancestor 12 million years ago. *Tripidium* is a monophyletic grouping composed of seven species, with a potential for the addition of two additional species, yet to be formally described. The inclusion of *Erianthus* sect *Ripidium* (which we re-classify as *Tripidium* species) within the ‘Saccharum complex’ has led sugarcane breeders down a blind alley for five decades and more, whilst breeders have ignored the potential of the New World *Erianthus* species, which do lie within the wild crossing window with sugarcane. Here we resolve the confusion and re-define the membership of the genus *Tripidium*, the ‘Saccharum complex’ and the Saccharinae based on robust molecular systematic studies.

## Materials and methods

### Plant materials and DNA isolation

*Tripidium arundinaceum* and *Tripidium ravennae* leaves were collected from the South African Sugarcane Research Institute’s (SASRI) collection, along with four other samples identified as *Tripidium*, but of previously unknown species. *Tripidium procerum*, *Tripidium rufipilum* and *Saccharum spontaneum* SES196 were from the USDA collection and DNA sequencing for these accessions has been reported previously [42]. For the SASRI accessions, total DNA was isolated from liquid nitrogen frozen and ground leaf material using the standard CTAB method according to Wang et al. [55]. Complete chloroplasts were amplified using the 13 primer pairs designed for this project (Additional file 1). Polymerase Chain Reaction (PCR) amplifications were performed using Phusion Hot Start II High-Fidelity DNA Polymerase (Thermo Scientific). Each reaction contained 50 ng of high quality genomic DNA, 1.5 mM MgCl_2_ with 0.2 mM each deoxynucleotide triphosphates (dNTPs), 0.5 M each of forward and reverse primer, 0.4 Units Phusion Hot Start II High-Fidelity DNA Polymerase and reaction buffer as supplied by the manufacturer. Thermal cycling conditions were as follows: Initial denaturation at 98 °C for 1 min, followed by 35 cycles of: 98 °C for 10 s and annealing and extension at 68 °C for 15 min. A final extension step was performed at 72 °C for 10 min followed by a hold at 4 °C. Amplicons were separated by gel electrophoresis (see example gel lanes, which are available in PDF format as an additional document Additional file 2), prior to gel elution and Illumina sequencing (Genotypic Technology Pvt. Ltd).

### Phenotypic characterization

Using Kew’s GrassBase [7] descriptions as a foundation, basic phenotypic characters for *T. arundinaceum, T. kanashiroi, T. bengalense, T. ravennae, T. procerum, T. rufipilium* and *T. strictum* were extracted. Potential *Tripidium* accessions from SASRI’s living collection were phenotypically characterized and matched against the above descriptions. Once identified, further characters were taken from SASRI’s accessions for *T. arundinaceum, T. kanashiroi* and *T. ravennae*. Further analysis of *Tripidium strictum* was made against the Missouri Botanical Garden (MBG) specimen of *Saccharum strictum* (MO-2397278). For *T. bengalense*, *T. rufipilum* and *T. procerum* the Herbarium Kewense (K) specimens of *Saccharum bengalense* (K000943381), *S. rufipilum* (K000309025) and *S. procerum* (K001128357) were analysed. Unique characters were used to generate a key to recognition of *Tripidium* species.

### Chloroplast assembly

The sequences derived from three USDA accessions were assembled as described previously [1] with data downloaded from NCBI’s sequence read archive (SRA) for the accessions: SRR2899231; SRR2891248 and SRR2891271, using Mirabait [56] and SPAdes v 3.10 [57], with a baiting k-mer of 27 and an assembly k-mer series of 25, 33, 55 and 77. Scaffolds were arranged against the previously published assembly of *Erianthus arundinaceus* JW63 (NCBI accession LC160130.1) and the region corresponding to the second inverted repeat was copied, inverted and stitched into the genome (that this region represented a repeat was confirmed by increased read coverage compared with the remainder of the genome). The SASRI accession raw read data were subject to adapter trimming and cleaning with Trimmomatic [58]. Trimmed reads were assembled, typically generating seven or eight scaffolds, which could be arranged on the *Erianthus arundinaceus* backbone. Any small gaps in the initial assemblies were filled by excising a 2 kb region around the gap and re-assembling this region by baiting reads with Mirabait (k-mer of 31) and assembling the baited reads with SPAdes before inserting the reassembled region to close the gap in the main assembly.

### Low copy number locus assembly

Following Estep et al. [17], low copy number regions were assembled for the following genes: *Aberrant panicle organization1* (*apo1*), *Dwarf8* (*d8*), two exons (7 and 8) of *Erect panicle* 2 (*ep2*), and *Retarded palea 1* (*rep1*). We assembled these regions from the following GenBank sequence read archive (SRA) accessions: *Sorghum propinquum* (Knuth) Hitchc. (SRR072055); *Sarga versicolor* (Andersson) Spangler (SRR427176); *Sarga timorense* (Kunth) Spangler Gypsum 9 (SRR424217); *Miscanthidium junceum* (Stapf) Stapf (SRR396848 and SRR396849); *Miscanthus sacchariflorus* (Maxim.) Benth. & Hook. f. ex Franch. var. Hercules (SRR486748); *Miscanthus x giganteus* JM Greef & Deuter ex Hodk. & Renvoize (SRR407328 and SRR407325); *Miscanthus transmorrisonensis* Hayata (SRR396850); *Saccharum* hybrid hort. ex RM Grey LCP85–385 (SRR427145); *Saccharum* hybrid SP80–3280 (SRR1763296), *Saccharum spontaneum* L. SES234B (SRR486146), *Sorghum bicolor* (L.) Moench BTx623 (SRR1945055) and *Sorghum arundinaceum* (Desv.) Stapf PI300119 (SRR999023). *Miscanthus sinensis* Andersson cv. Andante and *Andropogon virginicus* L. genomic reads as well as Sanger sequenced low copy number reads for *Tripidium arundinaceum* 2 were kindly donated by BeauSci, Cambridge, UK. For *Tripidium procerum* (ibid) and *Tripidium rufipilum* (ibid), as the data was low coverage, reads were mapped to the existing *Tripidium ravennae* gene fragments with BWA [59] prior to consensus sequence calling with the Integrative Genomics Viewer (IGV) [60]. The consensus sequences were used as templates for gene assembly in these sequences. Gene loci were assembled as described previously [61] using the closest orthologue for each gene for Mirabaiting and SPAdes for assembly. In *Miscanthus*, *M. sinensis* was used to bait the primary or ‘A’ genome for each species. All *Miscanthus sinensis* cv Andante, *Andropogon virginicus* assemblies along with the *T. rufipilum* and *T procerum* assemblies and the *Tripidium arundinaceum* 2 sequences have been deposited in EMBL/GenBank under the project identifier PRJEB22229. These and all other gene region assemblies were also deposited in the Dryad digital repository [62].

### Assembly finishing

To ensure that the assembly was of high quality, all assembled chloroplasts were finished and polished with a novel pipeline. Raw reads from the SRA pool were mapped back to the assembly with BWA [59], duplicate sequences were tagged with Picard tools [63] prior to optimizing the read alignment with GATK [64] and finally polishing and finishing with Pilon 1.2.0 [65].

### Chloroplast annotation

The finished chloroplast alignments were orientated so that they finished with the IR_B_ region prior to batch upload to the Verdant annotation server [66] for automated annotation. Annotation files were downloaded from Verdant in ‘.tbl’ (Verdant native) format. Genes and pseudogenes identified from our previous analysis [1], and which were not present in the Verdant annotation, were mapped manually with BLAST and were placed in a separate ‘.tbl’ format file. A custom BioPerl script [67] was written that integrated the Verdant output and the additional gene output along with data corresponding to gene annotation and cultivar level taxonomies prior to outputting an EMBL format file and a chromosome file that could be automatically uploaded to the EMBL database. All assembled chloroplasts were submitted to ENA under the project identifier PRJEB20532.

### Whole chloroplast alignments

Whole plastid alignments were performed with SATÉ (version 2.2.2) [68], using MAFFT [69] as the aligner, MUSCLE [70] as the sub-alignment joiner and RAxML [71] as the phylogenetic analysis application. Alignment iterations were run for 20 generations past the iterations that yielded the best likelihood score to ensure that the correct global alignment minimum had been reached. This optimal alignment was subsequently edited manually to remove any obvious errors and to trim all gaps of > 20 bp due to only a single sequence to 10 bp (a list of all chloroplast assemblies with sequence and voucher accessions are given in [Additional file 3]). This alignment was used to generate an initial phylogeny (corresponding to [Additional file 4]), but the raw alignment was used for the alignment finishing steps.

### Gene region alignments

Gene regions assembled in this study were merged with the equivalent gene regions from a subset of the data from Welker et al. [16]. All regions were aligned independently using SATÉ, with MAFFT as the aligner and Muscle as the sub-alignment joiner. Alignments were adjusted manually and trimmed. Each individual alignment was input into RAxML and a phylogenetic analysis (100 replicates) was run to identify the most likely tree. If alternate copies of genes were in the same position in the phylogeny these were linked. As we were not analysing close hybrids in this study, only primary copies of each locus were retained. Subsequent to alignment optimization (see below) individual gene/locus alignments were stitched together with a custom Perl script. If the positions of genes were uncertain, all alternate positions were generated. The phylogeny was run with RAxML and the sub-alignment giving the strongest phylogenetic signal was chosen. Using this methodology, we generated the optimal alignment. For the phylogeny generated here, only the alignment yielding the dominant phylogenetic signal was chosen and secondary copies of genes were eliminated. The final, optimized, alignment as well as the phylogram determined from this alignment were deposited in TreeBase (TB2: S23649).

### Alignment finishing and optimization

Both the whole chloroplast and individual gene locus alignments were finished and optimized using Prank [72], an indel aware probabilistic multiple alignment program. Terminal taxa representing well-supported groups as defined by SATÉ's RAxML phylogram were constrained using PRANK’s ‘group’ functions. The ‘alignment finishing’ mode of Prank was initiated with the following command:

prank -d=<input_alignment> -t=<input_tree> -o=<output_alignment> -partaligned

The output alignment was edited to remove any obviously over-inserted gaps and RAxML (see below) was run for 100 generations to generate a most likely phylogeny. This phylogeny and the manually fixed alignment were used as input for a second round of Prank analysis. After this round of analysis, as the input and output alignments yielded the same tree topology, Prank optimization was deemed complete. Finally, to reduce long-branch issues, all insertions of greater than 20 bp created by just a single sequence were trimmed to 20 bp. This final alignment was used for all subsequent analyses.

### Phylogenomic analyses

The whole plastid alignment was divided into LSC, IR_A_ and SSC partitions. These partitions were further divided into protein-coding gene, RNA-coding gene and non-coding regions. The regions were isolated with the BeforePhylo.pl [73] script and merged into separate partitions. The IR_A_ region contained only a single tRNA encoding gene, which was added to the SSC RNA-gene partition. This yielded a total of eight partitions. Best-fit evolutionary models for each partition were selected using JModelTest2 [74] and the AICc criterion. The best-fit models were as follows: LSC protein coding: TPM1uf + I + Γ, LSC RNA genes: TVM + Γ; LSC non-coding: TVM + Γ; IRA protein coding: TVM + I + Γ; IRA non-coding: TVM + Γ; SSC protein coding: TPM1uf + I + Γ; SSC RNA-gene: TrN + I + Γ and SSC non-coding: TVM + I + Γ. The low copy number gene loci were divided into their five component partitions. Each partition was tested with JModelTest2 and the AICc criterion to determine the best-fit evolutionary models. The optimal models were as follows: apo1: GTR + Γ; d8: GTR + Γ + I; ep2-exon7: TIM2 + G; ep2-exon8: TrN + Γ + I; rep1: HKY + G. The partitions determined above and their closest model equivalents were used for all subsequent analyses. Neighbor-joining phylogenies were generated with the Ape library in R [75]. Bayesian analyses were run with MrBayes (version 3.1.2) [76], Maximum Likelihood analyses were run with RAxML (version 8.1.17) [72] and SH-aLRT analyses were run with IQ-Tree [77].

For both the chloroplast and low copy number gene datasets Bayesian Markov Chain Monte Carlo (MCMC) analyses were run with MrBayes 3.1.2, using four chains (3 heated and 1 cold) with default priors run for 20,000,000 generations with sampling every 100th tree. Two independent MrBayes analyses, each of two independent runs, were conducted. To avoid any potential over-partitioning of the data, the posterior distributions and associated parameter variables were monitored for each partition using Tracer v 1.6 [78]. High variance and low effective sample sizes were used as signatures of over-sampling. Burn-in was determined by topological convergence and was judged to be sufficient when the average standard deviation of split frequencies was < 0.001 along with the use of the Cumulative and Compare functions of AWTY [79]. The first 50,000 (25%) sampled generations were discarded as burn-in, and the resultant tree samples were mapped onto the reference phylogram (as determined by maximum likelihood analysis) with the SumTrees 4.0.0 script of the Dendropy 4.0.2 package [80].

Maximum Likelihood inference and bootstrapping were performed in RAxML using the same partitioning schemes as detailed above. To obtain the best tree RAxML was run without resampled replicates for 100 generations. The most likely whole plastome tree was obtained in the 70th generation and the most likely low copy number gene locus tree was obtained on the 53rd generation. These were used as the respective reference tree topologies in all subsequent analyses. To confirm this topology, a second, independent run of RAxML with different seed parameters was also run for both data matrices. In all cases, replicate tree topologies were identical.

To provide support for the Maximum Likelihood phylogeny, a total of 10,000 bootstrap replicates were analysed. Replicate trees were summarized with SumTrees before being mapped onto the best maximally likely tree as determined above.

As an additional measure of branch confidence, SH-aLRT analyses were run for 2500 replicates with IQ-TREE, using the -bnni option [77] to reduce the risk of overestimating branch supports due to severe model violations.

### Divergence time estimates

Divergence times were estimated using BEAST 2.4.4 [81] optimized for OpenGL graphics running on a MacBook Pro (15-in., 2017 2.9 GHz Intel Core i7) with 16Gib RAM. The concatenated analysis (LSC, IRA and SSC along with their coding gene, RNA gene and non-coding partitions) was run for 20 million generations with sampling every 1000th replication under the BEAST equivalents of the JModelTest2 models (as defined above) with six gamma categories. The tree prior used the Calibrated Yule Model [81] with a relaxed lognormal clock and site models unlinked. Partitions were defined as above. The XML output from BEAUTi was edited to set the starting tree as the most likely tree obtained from RAxML analysis. The site model followed an uncorrelated lognormal relaxed clock [82]. The whole chloroplast analysis was rooted to *Arundinella deppeana,* whilst the low copy number gene locus analysis was rooted to *Arthraxon prionodes* and *Arthraxon lanceolatus*. The age of *Zea mays* divergence was estimated as a normal distribution describing an age of 13.8 ± 2 million years [17] whilst the age of the root was set as 24 ± 4 million years for the chloroplast phylogeny and 19 ± 4 million years for the low copy number gene loci analyses, both based on prior analyses (D Lloyd Evans, personal communication). Convergence statistics were estimated using Tracer v.1.6 [78] after a burn-in of 20,000 sampled generations. Chain convergence was estimated to have been met when the effective sample size was > 200 for all statistics. Tree samples were integrated with SumTrees to generate the maximum clade credibility tree and to determine the 95% highest posterior density (HPD) for each node. The final tree was drawn using FigTree v.1.4.3 [83].

The final alignments and phylogenies are available from TreeBase [84].

## Additional files

Additional file 1: List of *Tripidium* chloroplast amplification primers. List of the 13 primers used in amplifying the complete chloroplast sequence of the South African Sugarcane Research Institute *Tripidium* accessions. (PDF 58 kb)

Additional file 2: Gel images of PCR amplicons. Gel images of the 13 PCR amplicons used for *Tripidium* chloroplast isolation and assembly. Example images for the 13 primers are shown, with *Saccharum* hybrid BH10/12 as a positive control. There are images for all six of the *Tripidium* accessions from the South African Sugarcane Research Institute sequenced and assembled in this study. (PDF 311 kb)

Additional file 3: Table of whole chloroplast accessions used for phylogenetics. A table of all the chloroplast sequence accessions (including species, voucher accession and ENA/GenBank accession) that were used for the phylogenetic analyses in this study. Also given are the original references (where applicable) for each sequence. (PDF 150 kb)

Additional file 4: Phylogram with support values for a traditional whole chloroplast analysis. The image depicts the most likely tree topology (with branch support) for an analysis of a whole chloroplast alignment using a standard partition of LSC, IR_A_ and SSC. Numbers next to nodes give support values (non-parametric bootstrap/Bayesian inference). (PDF 110 kb)

## Abbreviations

bp: basepairs
cv.: cultivar
Mya: million years ago
*s.s*.: *sensu stricto*
sp.: species
