# Supplementary material for: Whole chloroplast genome and gene locus phylogenies reveal the taxonomic placement and relationship of Tripidium (Panicoideae: Andropogoneae) to sugarcane
Source: BMC Evol Biol. 2019 Jan 25;19:33. doi: 10.1186/s12862-019-1356-9 (PMC6347779; doi:10.1186/s12862-019-1356-9)
Supplement: Supplementary file 1 — List of Tripidium chloroplast amplification primers. List of the 13 primers used in amplifying the complete chloroplast sequence of the South African Sugarcane Research Institute Tripidium accessions. (PDF 58 kb) [file 12862_2019_1356_MOESM1_ESM.pdf]

Additional file 1

Primer Set	Forward	Reverse	Expected amplicon size	
1	ATTTGCGGGTTCAATTCCTGCTGGATG	ATGGCTGAGTGGACTAAAGCGGCGGATT	11003	
2	AAGAGAGGGATTCGAACCCTCGGTACAA	CTTGACAGGGCGGTGCTCTGACCAAT	8613	
3	AGTGGTTCAGGACATCTCTCTTTCAAG	GAAGTAGCACGTTTCGTGATTAAATC	8888	
4	GATTTAATCACGAAACGTGCTACTTC	AGCTGTTTGGTAGCTCACAAGGCTCAT	12631	
5	CCCCAAGGTTATGAGCCTTGTGAGCTA	CTACATAGCAGTTCCAATGCTACGCCT	8329	
6	ATGTAGGAGAGATGGCCGAGCG	TTGAACCAATGACTCCCGCCGTAT	7272	
7	ACGGCGGGAGTCATTGGTTCA	AGTTCGGTAGAACGTGGGTCTCCA	11951	
8	TGAACCTACGACATCGGGTTTTGGAGA	AACAAGTAAGTGTAATTCCCCCTAAA	8174	
9	GCTTGAGCCGTACGAGATGAAATTTT	AGAGCGTGGAGGTTCGAGTCC	14829	
10	CTCGAACCTCCACGCTCT	GCCGCTACTCGGACTCGAACC	20371	
11	CTCGAACCTCCACGCTCT	CATCCTGGACTTGAACCAGAGACC	9384	
12	GGTTCGAATCCGAGTGGCGGC	AGCACGTGGCTACGAACCACGGT	Primary amplicon: 11099	Secondary amplicon: 17200
13	GATCCAGTGGAGACGGGGTGgg	ACCAATTTCACCATGGCGGCta	Primary amplicon: 6169	Secondary amplicon: 17344

Table showing the primers used to amplify the *Tripidium* accessions from the SASRI (South African Sugarcane Research Institute) collection. The primers are shown mapped to the *Tripidum arundinaceum* chloroplast genome in Figure 1 of the main manuscript. Gel images of all the amplicons generated from these primers are shown in Additional file 2.
