# Supplementary material for: Whole chloroplast genome and gene locus phylogenies reveal the taxonomic placement and relationship of Tripidium (Panicoideae: Andropogoneae) to sugarcane
Source: BMC Evol Biol. 2019 Jan 25;19:33. doi: 10.1186/s12862-019-1356-9 (PMC6347779; doi:10.1186/s12862-019-1356-9)
Supplement: Supplementary file 2 — Gel images of PCR amplicons. Gel images of the 13 PCR amplicons used for Tripidium chloroplast isolation and assembly. Example images for the 13 primers are shown, with Saccharum hybrid BH10/12 as a positive control. There are images for all six of the Tripidium accessions from the South African Sugarcane Research Institute sequenced and assembled in this study. (PDF 311 kb) [file 12862_2019_1356_MOESM2_ESM.pdf]

**Additional file 2**

Primer set 1: Expected size 11003bp

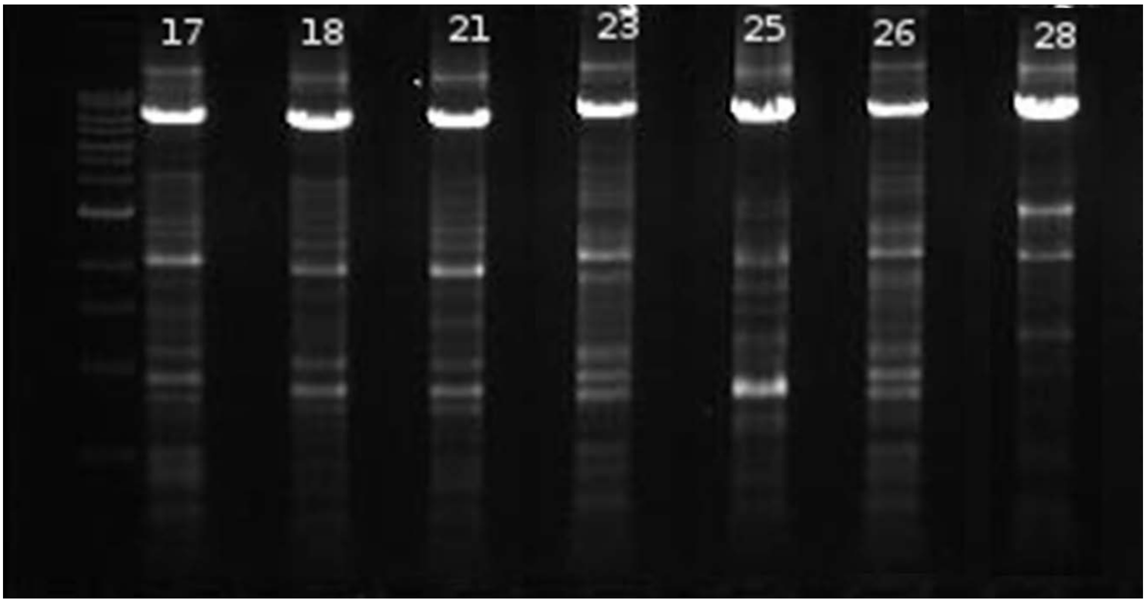


Primer set 2: Expected size 8613bp

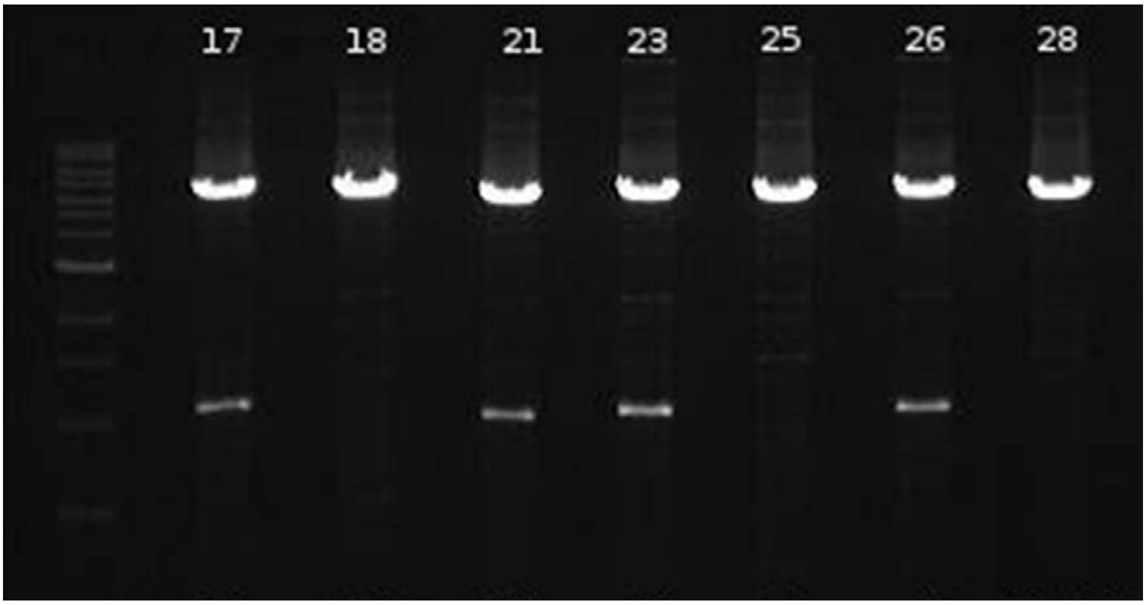

Primer set 3: Expected size 8888bp

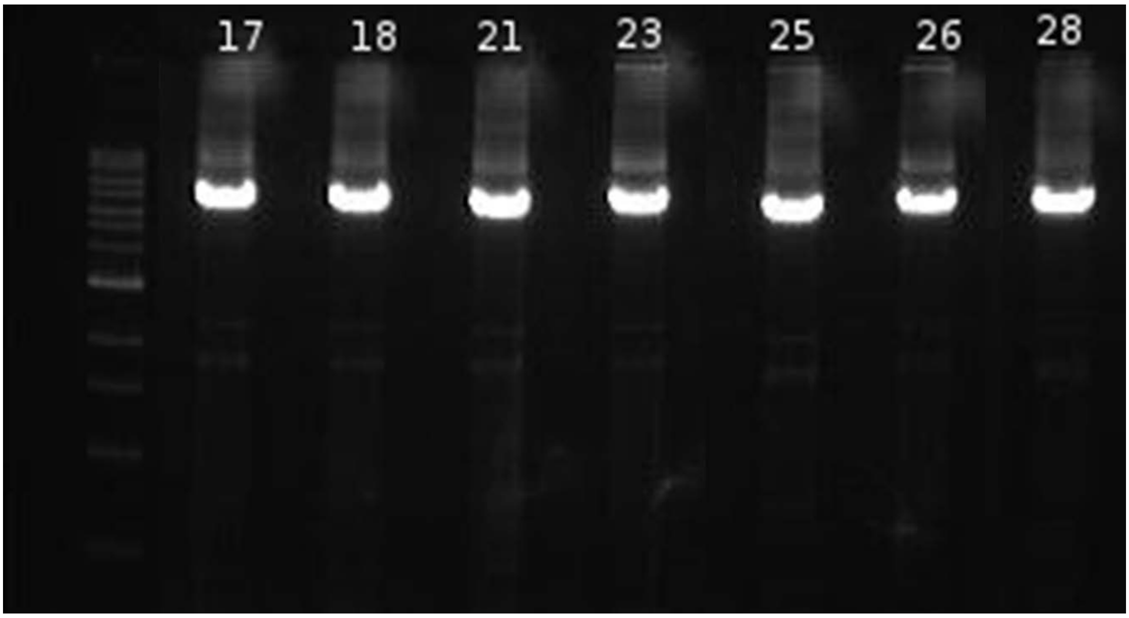


Primer set 4: Expected size 12631bp

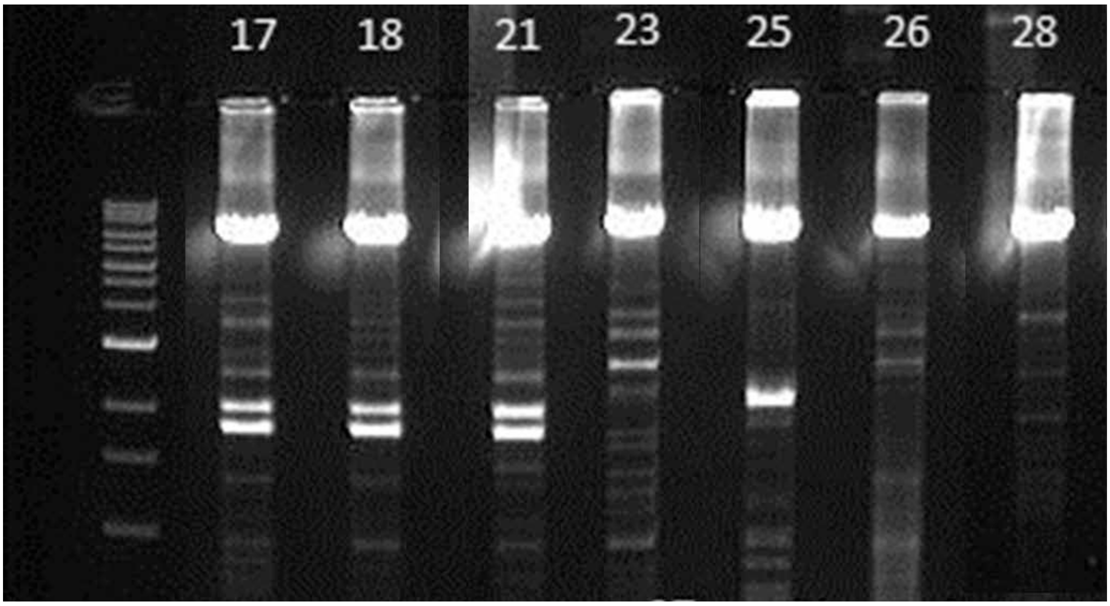

Primer set 5: Expected size 8329bp

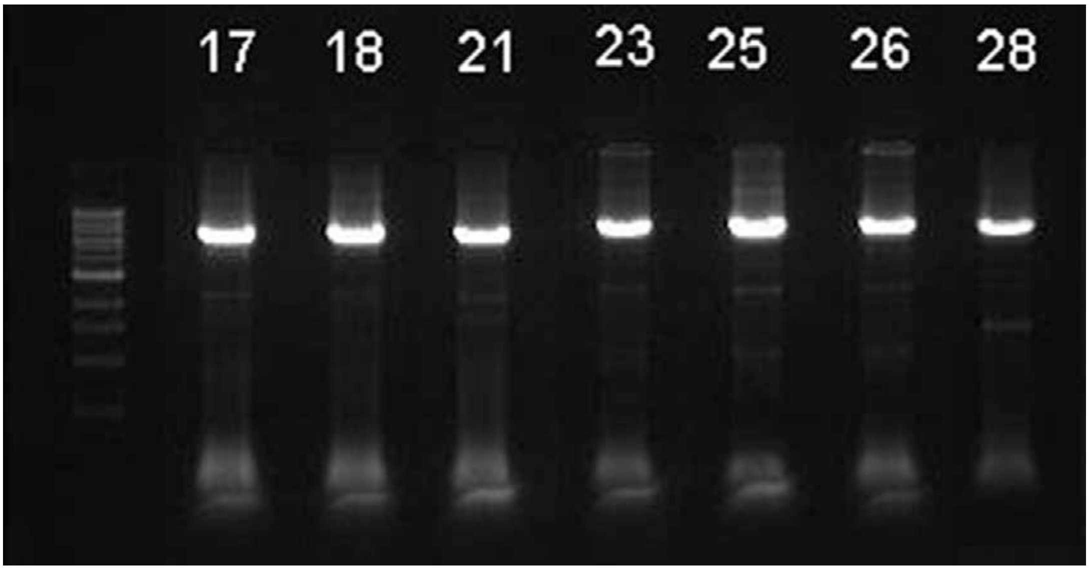


Primer set 6: Expected size 7272bp

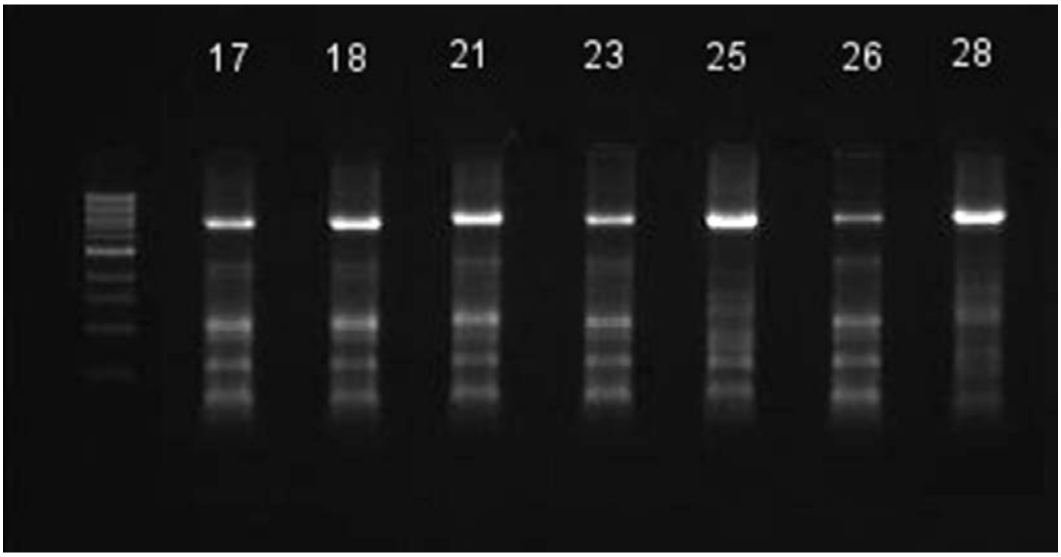

Primer set 7: Expected size 11951bp

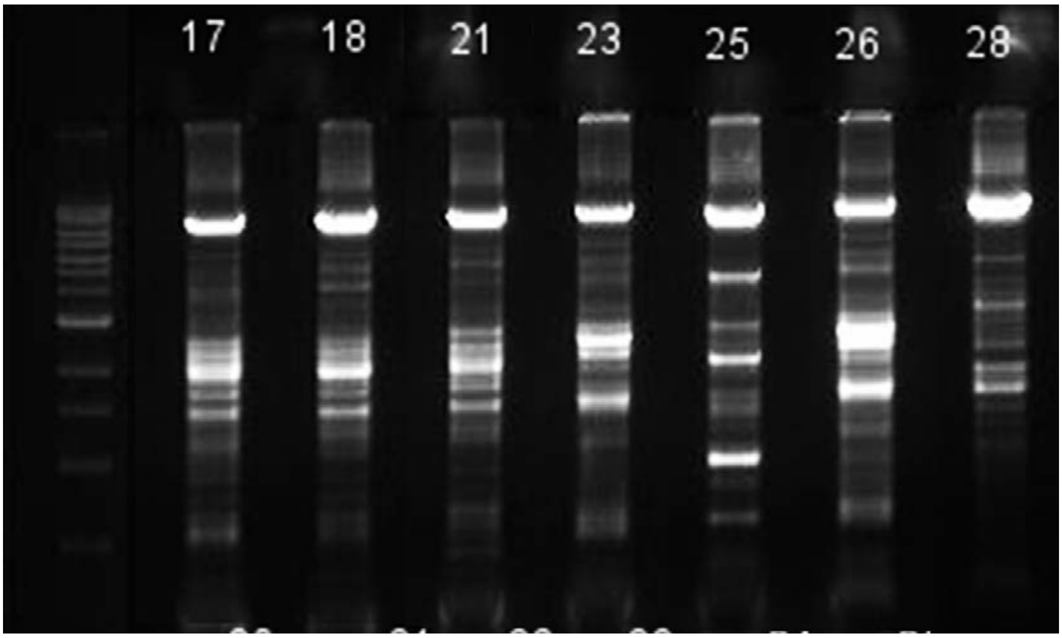


Primer set 8: Expected size 8174bp

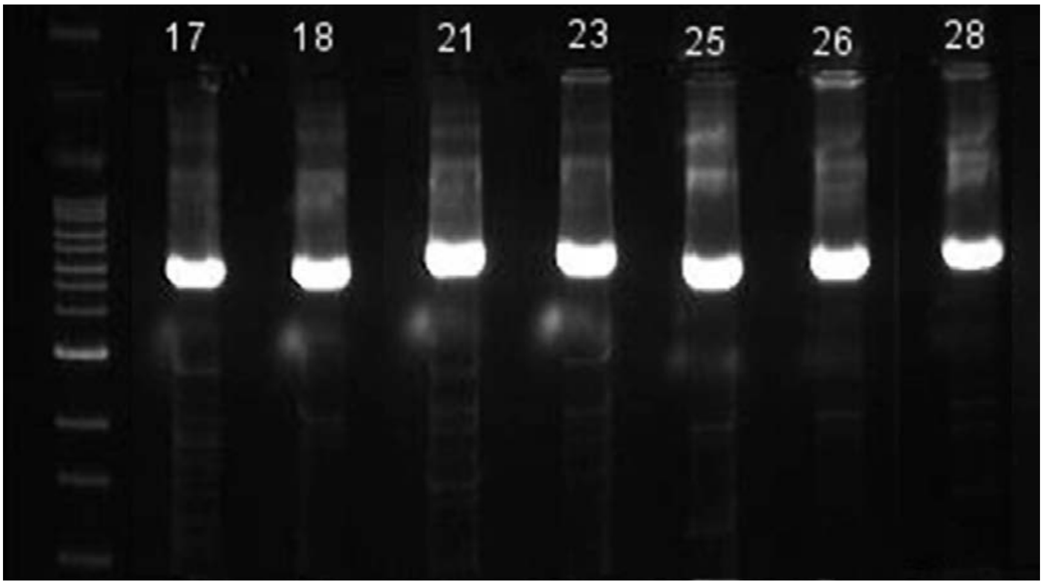

Primer set 9: Expected size 14829bp

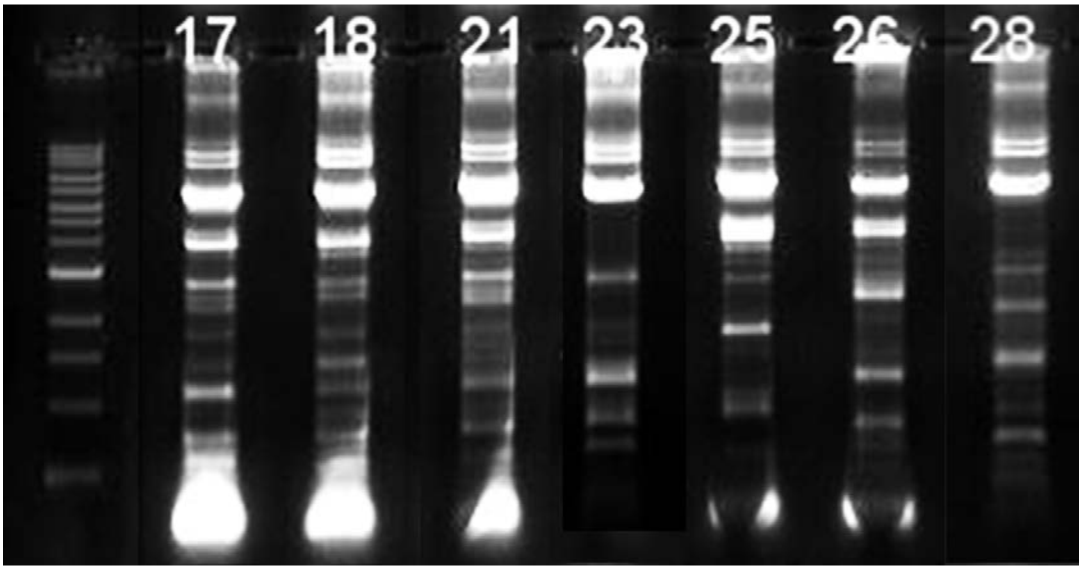


Primer set 10: Expected size 20371bp

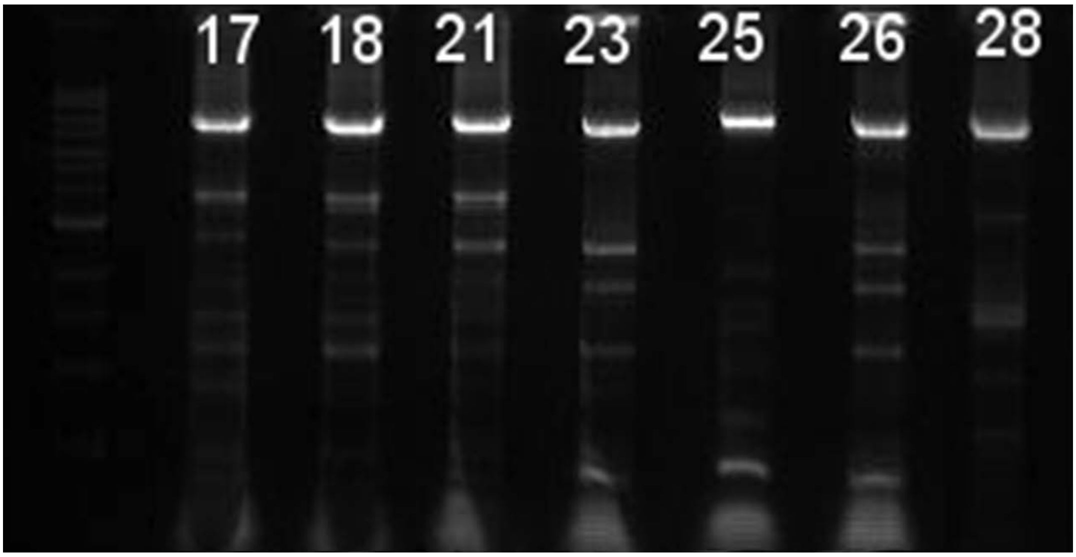

Primer set 11: Expected size 9384bp

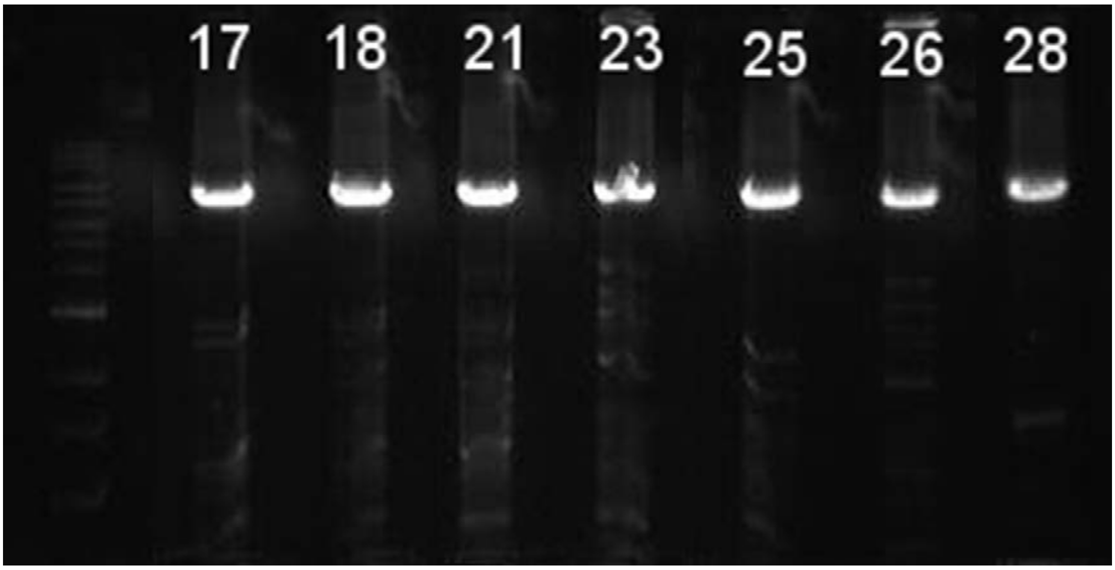


Primer set 12: Expected size 11099bp

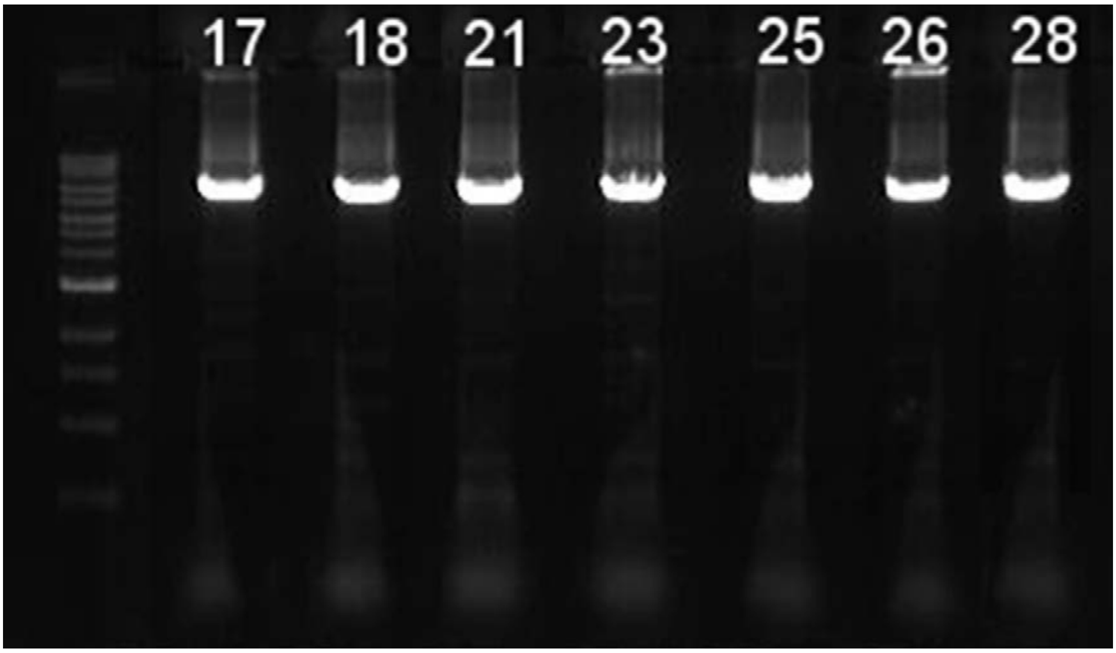

Primer set 13: Expected size 6169bp

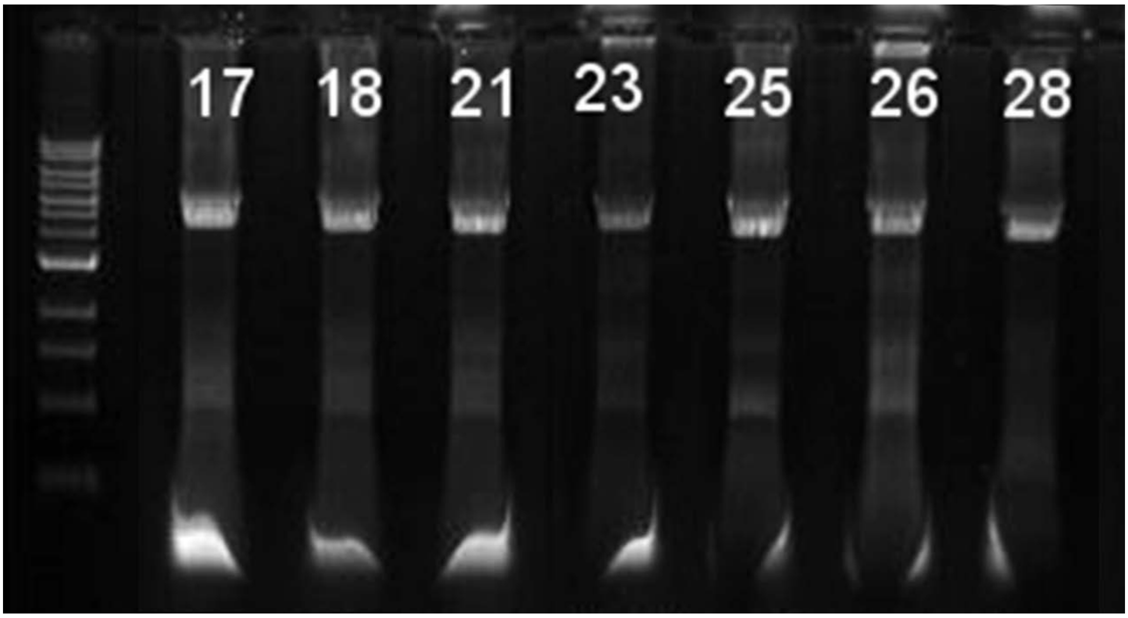


Image manipulation: Original images were stored as .jpg. The images were upscaled to 300dpi, a standard Photoshop filter was applied to remove JPEG artifacts. Contrast was improved and balanced using Photoshop's 'Adjust Contrast' tool. As only Tripidium accessions and the reference Saccharum officinarum BH10/12 cultivar were of interest, these lanes were excised from a larger gel. Amplicons are labelled 1 to 13 as in Supplementary Table 1 and Figure 1 of the main article. Lanes are labelled as follows

Lane 1 (not numbered) 1 kbp ladder

17 *Tripidium arundinaceum* SA-E1

18 *Tripidium kanashiroi* SA-E2

21 *Tripidium* sp. NG77-188

23 *Tripidium arundinaceum* IK76-417

25 *Tripidium ravennae*

26 *Tripidium arundinaceum*

28 *Saccharum* hybrid BH10/12

In all cases, the appropriate amplicon band was excised from the gel prior to elution, purification, lyophilisation and sequencing.
