# Supplementary material for: Whole chloroplast genome and gene locus phylogenies reveal the taxonomic placement and relationship of Tripidium (Panicoideae: Andropogoneae) to sugarcane
Source: BMC Evol Biol. 2019 Jan 25;19:33. doi: 10.1186/s12862-019-1356-9 (PMC6347779; doi:10.1186/s12862-019-1356-9)
Supplement: Supplementary file 3 — Table of whole chloroplast accessions used for phylogenetics. A table of all the chloroplast sequence accessions (including species, voucher accession and ENA/GenBank accession) that were used for the phylogenetic analyses in this study. Also given are the original references (where applicable) for each sequence. (PDF 150 kb) [file 12862_2019_1356_MOESM3_ESM.pdf]

Additional file 3

Species	Voucher or Accession	GenBank Accession	Reference
Arundinella deppeana Nees ex Steud.	XAL:Clark et al. 1680	KU291490.1	Burke et al. 2016
Arthraxon prionodes (Steud.) Dandy	PI<ITA>:659331	KU291471.1	Burke et al. 2016
Chrysopogon serrulatus Trin.		KU961864.1	Welker et al. 2016
Eriochrysis laxa Swallen		KU961863.1	Welker et al. 2016
Eriochrysis villosa Swallen		KU961860.1	Welker et al. 2016
Saccharum spontaneum L.	SES205A	LN896360.1	Lloyd Evans and Joshi 2016
Saccharum spontaneum L.	SES234B	LN849912.1	Lloyd Evans, D. (submitter)
Saccharum hybrid cultivar hort. Ex RM Grey	Q165	LN896359.1	Lloyd Evans and Joshi 2016
Saccharum hybrid cultivar hort. Ex RM Grey	SP80-3280	AE009947.2	Calsa Jr et al. 2004
Saccharum hybrid cultivar hort. Ex RM Grey	NCo310	AP006714.1	Asano et al. 2004
Saccharum officinarum L.	IJ76-514	LN849913.1	Lloyd Evans and Joshi 2016
Miscanthidium junceum Stapf (Stapf)		LN869216.1	Lloyd Evans, D. (submitter)
Miscanthidium capense (Nees) Stapf	Joshi 1	???????	Lloyd Evans, D. (submitter)
Miscanthus sacchariflorus (Maxim.) Benth. & Hook. f. ex Franch.	cv Hercules	LN869218.1	Lloyd Evans, D. (submitter)
Miscanthus sinensis Andersson	cv Andante	ERS2073004	Lloyd Evans, D. (submitter)
Miscanthus floridulus (Labill.) Warb. Ex K Schum. & Lauterb.	PI295762	LN869215.1	Lloyd Evans, D. (submitter)
Sarga timorense Lloyd Evans		KF998272.1	Kepers et al. (submitter)
Sorghum bicolor (L.) Moench	BTx623	EF115542.1	Saski et al. 2007
Imperata cylindrical (L.) Raeusch.	DEK:Burke 21	KU291466.1	Burke et al. 2016
Pogonatherum paniceum (Lam.) Hack.		KU961859.1	Welker et al. 2016
Eulalia aurea (Bory) Knuth	PI<ITA>:12153	KU291499.1	Burke et al. 2016
Sorghastrum nutans (L.) Nash	DEK:Wysocki s.n.	KU291482.1	Burke et al. 2016
Hyparrhenia subplumosa Stapf	PI<ITA>:12665	KU291500.1	Burke et al. 2016
Diheteropogon amplectens Nees (Clayton)	var. catangensis voucher PI<ITA>:12585	KU291497.1	Burke et al. 2016
Themeda sp. Forssk.	Saarela 1833	KU291484.1	Burke et al. 2016
Iseilema macratherum Domin	PI<ITA>:257760	KU291468.1	Burke et al. 2016
Capillipedium venustum (Thwaites) Bor	PI<ITA>:11713	KU291493.1	Burke et al. 2016
Bothriochloa alta (Hitchc.) Henrard	DEK:Duvall s.n.	KU291492.1	Burke et al. 2016
Tripidium arundinaceum Lloyd Evans	JW630	LC160130.1	Tsuruta et al. 2016
Ischaemum afrum (JF Gmel.) Dandy	PI<ITA>:364924	KU291467.1	Burke et al. 2016
Rottboellia cochinchinensis (Lour.) Clayton	ISC<USA-IA>:Clark et al. 1698	KU291481.1	Burke et al. 2016
Coix lacryma-jobi L.	M. Duvall s.n. 26May 2006 (DEK)	FJ261955.1	Leseberg and Duvall 2009

Dimeria ornithopoda Trin.		KY596130.1	Arthan et al. 2017
Eulaliopsis binate (Retz.) CE Hubb.		KY596182.1	Arthan et al. 2017
Heteropogon triticeus (R. Br.) Stapf ex Craib		KY596142.1	Arthan et al. 2017
Andropogon distachyos L.		KY596170.1	Arthan et al. 2017
Schizachyrium sanguineum (Retz.) Alston		KY596124.1	Arthan et al. 2017
Hemisorghum mekongense (A. Camus) CE Hubb.		KY596132.1	Arthan et al. 2017
Eremochloa ciliaris (L.) Merr.		KY596146.1	Arthan et al. 2017
Mnesithea helferi (Hook. f.) de Koning & Sosef		KY596162.1	Arthan et al. 2017
Sorghum propinquum (Knuth) Hitch.	369-1	?????	Lloyd Evans, D. (submitter)
Zea mays L.	B73	AY928077.1	Schnable et al. 2009
Zea luxurians (Durieu & Asch.) RM Bird		KR873424.1	Orton 2015

Arthan W, McKain MR, Traiperm P, Welker CA, Teisher JK and Kellogg EA. 2017. Phylogenomics of Andropogoneae (Panicoideae: Poaceae) of Mainland Southeast Asia. Systematic Botany, 42:418-431.

Asano T, Tsudzuki T, Takahashi S, Shimada H, Kadowaki KI. 2004. Complete nucleotide sequence of the sugarcane (Saccharum officinarum) chloroplast genome: a comparative analysis of four monocot chloroplast genomes. DNA Research 11:93–99.

Burke, S.V., Wysocki, W.P., Zuloaga, F.O., Craine, J.M., Pires, J.C., Edger, P.P., Mayfield-Jones, D., Clark, L.G., Kelchner, S.A. and Duvall, M.R., 2016. Evolutionary relationships in Panicoid grasses based on plastome phylogenomics (Panicoideae; Poaceae). *BMC Plant Biology*, *16*(1), p.140.

Calsa Jr T, Carraro DM, Benatti MR, Barbosa AC, Kitajima JP, Carrer H. 2004. Structural features and transcript-editing analysis of sugarcane (Saccharum officinarum L.) chloroplast genome. Current Genetics 46:366–373.

Leseberg CH, Duvall MR. 2009. The complete chloroplast genome of Coix lacryma-jobi and a comparative molecular evolutionary analysis of plastomes in cereals. Journal of Molecular Evolution 69:311-318.

Lloyd Evans D, Joshi SV. 2016. Complete chloroplast genomes of Saccharum spontaneum, Saccharum officinarum and Miscanthus floridulus (Panicoideae: Andropogoneae) reveal the plastid view on sugarcane origins. Systematics and Biodiversity, 14:548–571.

Orton, L.M., 2015. *Phylogenomic study of selected species within the genus Zea: Mutation rate analysis of complete chloroplast genomes* (Doctoral dissertation, Northern Illinois University).

Saski C, Lee SB, Fjellheim S, Guda C, Jansen RK, Luo H, Tomkins J, Rognli OA, Daniell H, Clarke JL. 2007. Complete chloroplast genome sequences of Hordeum vulgare, Sorghum bicolor and Agrostis stolonifera, and comparative analyses with other grass genomes. Theoretical and Applied Genetics 115:571–590.

Schnable PS, Ware D, Fulton RS, Stein JC, Wei F, Pasternak S, Liang C, Zhang J, Fulton L, Graves TA, Minx P. 2009. The B73 maize genome: complexity, diversity, and dynamics. Science 326:1112–1115.

Tsuruta SI, Ebina M, Kobayashi M, Takahashi W. 2017. Complete Chloroplast Genomes of Erianthus arundinaceus and Miscanthus sinensis: Comparative Genomics and Evolution of the Saccharum Complex. PloS One, 12:e0169992.

Welker CA, Souza-Chies TT, Longhi-Wagner HM, Peichoto MC, McKain MR, Kellogg, EA. 2016. Multilocus phylogeny and phylogenomics of Eriochrysis P. Beauv.(Poaceae–Andropogoneae): Taxonomic implications and evidence of interspecific hybridization. Molecular Phylogenetics and Evolution 99:155–167.

Note to reviewers, accessions with ???? have been submitted to ENA and have been validated, but we do not yet have accessions. These will be updated in the next version of the manuscript.
