# Supplementary material for: Whole chloroplast genome and gene locus phylogenies reveal the taxonomic placement and relationship of Tripidium (Panicoideae: Andropogoneae) to sugarcane
Source: BMC Evol Biol. 2019 Jan 25;19:33. doi: 10.1186/s12862-019-1356-9 (PMC6347779; doi:10.1186/s12862-019-1356-9)
Supplement: Supplementary file 4 — Phylogram with support values for a traditional whole chloroplast analysis. The image depicts the most likely tree topology (with branch support) for an analysis of a whole chloroplast alignment using a standard partition of LSC, IRA and SSC. Numbers next to nodes give support values (non-parametric bootstrap/Bayesian inference). (PDF 110 kb) [file 12862_2019_1356_MOESM4_ESM.pdf]

Additional file 4

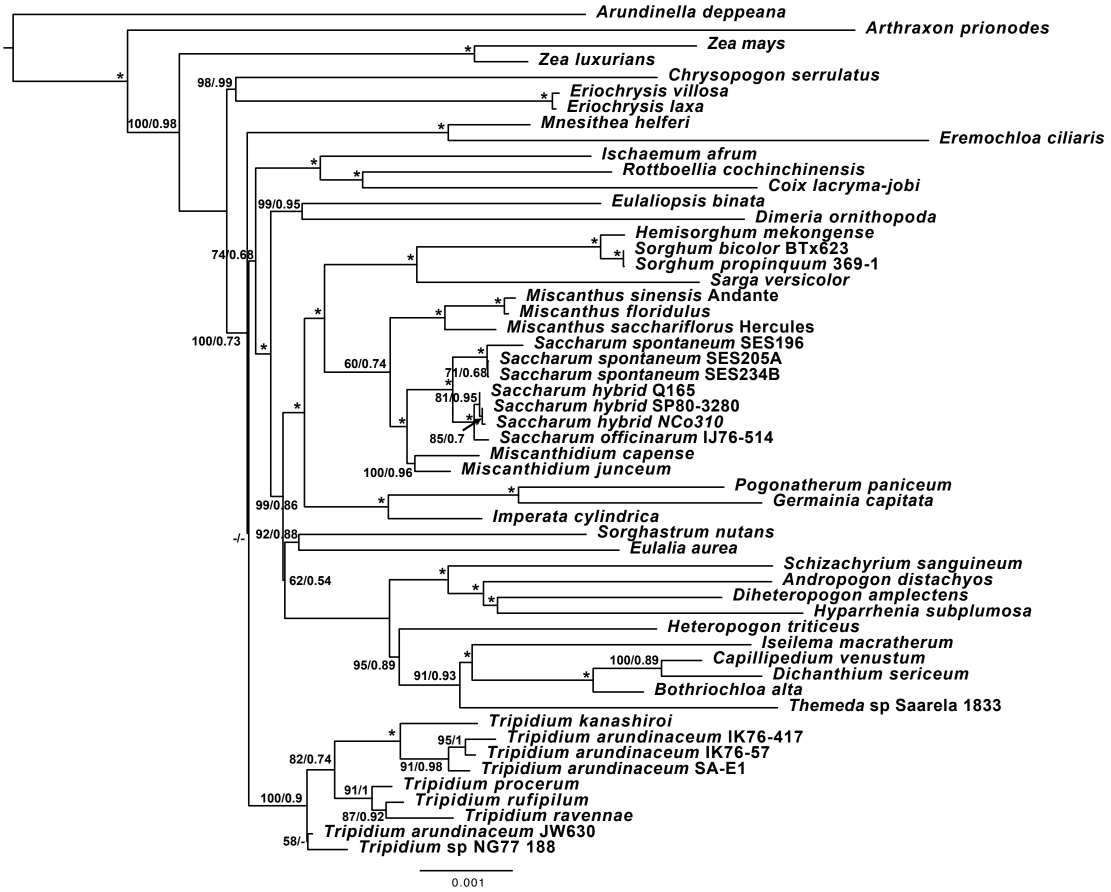

Phylogeny generated from a standard alignment, after Prank optimization, using LSC, IR_A and SSC partitions only. Topological changes and generally poorer branch supports are shown so that this phylogeny can be compared with Figure 3 of the main manuscript. Branch supports are given as non-parametric bootstrap values/Bayesian inference values. The scale bar at the bottom represents the number of substitutions per site. The plylogeny was rooted on *Arundinella deppeana*.
